# Local thermoelectric response from a single Néel domain wall

**DOI:** 10.1126/sciadv.adc9798

**Published:** 2022-11-23

**Authors:** Robert Puttock, Craig Barton, Elias Saugar, Petr Klapetek, Alexander Fernández-Scarioni, Paulo Freitas, Hans W. Schumacher, Thomas Ostler, Oksana Chubykalo-Fesenko, Olga Kazakova

**Affiliations:** ^1^ National Physical Laboratory, Hampton Road, Teddington TW11 0LW, UK.; ^2^ Instituto de Ciencia de Materiales de Madrid, ICMM–CSIC, Campus de Cantoblanco, C. Sor Juana Inés de la Cruz, 3, Madrid 28049, Spain.; ^3^ Czech Metrology Institute, Okruzni 772/31, Brno 10135, Czech Republic.; ^4^ Central European Institute of Technology (CEITEC), Brno University of Technology, Purkynova 123, Brno 612 00, Czech Republic.; ^5^ Physikalisch-Technische Bundesanstalt, Bundesallee 100, 38116 Braunschweig, Germany.; ^6^ Instituto de Engenharia de Sistemas e Computadores (INESC-MN), R. Alves Redol 9, 1000-029 Lisboa, Portugal.; ^7^ Sheffield Hallam University, Howard Street, Sheffield S1 1WB, UK.; ^8^ Department of Physics and Mathematics, University of Hull, Cottingham Road, Hull HU6 7RX, UK.

## Abstract

Spatially resolved thermoelectric detection of magnetic systems provides a unique platform for the investigation of spintronic and spin caloritronic effects. Hitherto, these investigations have been resolution-limited, confining analysis of the thermoelectric response to regions where the magnetization is uniform or collinear at length scales comparable to the domain size. Here, we investigate the thermoelectric response from a single trapped domain wall using a heated scanning probe. Following this approach, we unambiguously resolve the domain wall due to its local thermoelectric response. Combining analytical and thermal micromagnetic modeling, we conclude that the measured thermoelectric signature is unique to that of a domain wall with a Néel-like character. Our approach is highly sensitive to the plane of domain wall rotation, which permits the distinct identification of Bloch or Néel walls at the nanoscale and could pave the way for the identification and characterization of a range of noncollinear spin textures through their thermoelectric signatures.

## Teaser

Spatially resolved thermoelectric response from single nanoscale magnetic spin texture reveals Néel domain-wall like character.

## INTRODUCTION

Spin caloritronics describes the interplay of spin with temperature and charge-based transport phenomena. It has emerged as an intensely researched area for both fundamental and physical understanding and shows promise for future technological applications. Substantial research has targeted “green” thermoelectric (TE) technologies for energy harvesting (*1*–*3*) and biotechnology (*4*–*6*), with emphasis placed on optimizing the figure of merit for energy conversion (*7*). As such, increasing efforts are focused on the application of spin caloritronic phenomena, such as spin Seebeck (*8*, *9*), spin Peltier (*10*, *11*), and the spin Nernst effect (*12*) in addition to thermal analogs of the Hall effect, which include the Righi-Leduc (*1*, *13*), Ettingshausen (*14*), and Nernst effects (*15*–*17*). Recently, TE transport phenomena have been used for the characterization of quantum materials (*18*, *19*), for thermal dissipation characterization in magnetic tunnel junctions (*20*), and for the detection of novel magnetic solitons, such as magnetic domain walls (*17*) and skyrmions (*21*, *22*).

Measurements of TE phenomena are typically performed by subjecting a macroscopic sample to a global thermal gradient from a resistive heater or using Peltier plates (*23*). However, this approach samples a large integrated response over all the TE effects that are present, which makes resolving the spatially varying magnetic state nontrivial. To circumvent this, laser excitation (*24*–*27*), near-field optical excitation (*28*, *29*), or heated scanning probes (*30*, *31*) have been used to generate microscopic hotspots. These methods have led to a better understanding of the underlying magnetic state at shorter length scales. However, these examples have not been able to adequately explain the intricate TE responses that may arise from regions where the magnetization is nonuniform (e.g., domain walls).

Here, we detect and separate the individual nanoscopic TE signatures of a domain wall pinned in an ultrathin Pt/Co_60_Fe_20_B_20_/Pt trilayer by localized scanning thermoelectric microscopy (sThEM). This improvement in nanoscopic TE imaging using a highly localized heat source and validation by thermal micromagnetic modeling provides new insights into spin caloritronic responses at nanometer length scales. We show how the highly localized generation of nonequilibrium spin-polarized currents in the vicinity of novel spin textures results in a significant TE response. We exemplify this by controllably trapping a Néel domain wall and spatially mapping the unique TE signature that arises at the nanometer scale.

Our experimental observations are thoroughly described through a combination of thermal modeling, analysis of analytic expressions, and micromagnetic modeling using the Landau-Lifshitz-Bloch (LLB) equation (*32*–*34*). The results from the LLB are subsequently used to calculate the TE response expected for an energetically minimized Néel domain wall configuration. We find that our experimental observations are well understood by considering the anomalous Nernst effect (ANE) in combination with the planar Nernst effect (PNE) and anisotropic magnetothermopower (AMTP). We show that the responses from Bloch and Néel domain walls are distinguishable, demonstrating the ability to discern domain wall type by this experimental method. The difference in Néel- and Bloch-type signatures may also be applied to other nanoscale topological spin textures.

The ability to directly resolve the nanoscale magnetization is important to address the underconstrained problem of determining the spin texture, where a manifold of potential solutions can exist (*35*). This is required to accelerate material processing and device development for next-generation green technologies.

### Symmetry of magnetothermoelectric effects

The geometrical relationship between the TE effects can be constructed through the Seebeck tensor *S*, which is analogous to the anisotropic magnetoresistance tensor (*17*, *36*). Hence, the electric field $\overset{⇀}{E}$ that results from the ANE, PNE, and AMTP is described by the following relationship for the general case where the magnetization $\hat{m}$ points along an arbitrary direction

$$E=S_{⊥}\nabla T+(S_{∥}-S_{⊥})(\nabla T∙\hat{m})\hat{m}-S_{N}(\hat{m}\times \nabla T\;)$$ (1)

**Fig. 1.**
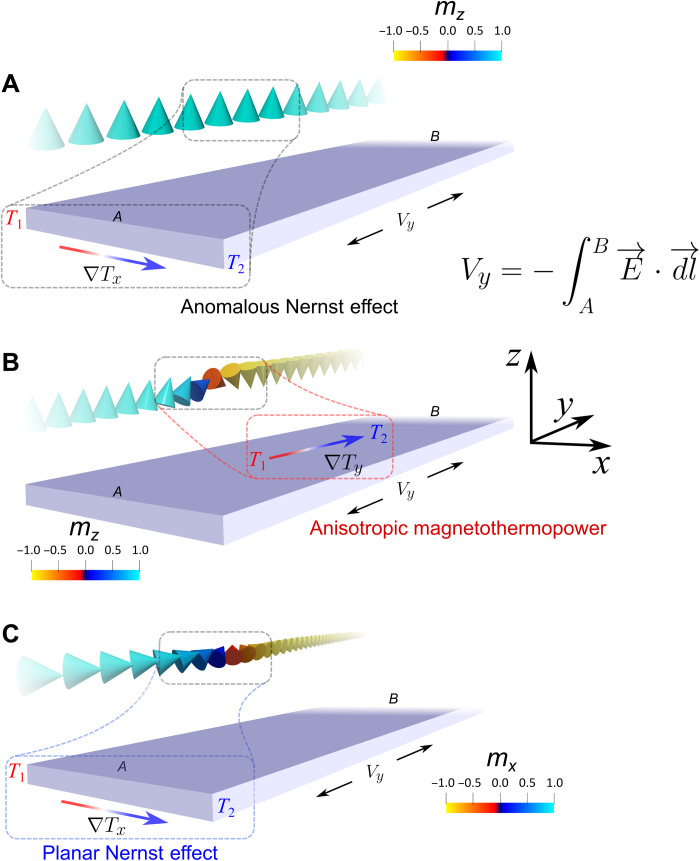
Overview of the TE effects in an ultrathin magnetic PMA nanowire. (**A**) The anomalous Nernst effect (ANE), where an ANE voltage along *y* will arise when a nonzero *z* component of the magnetization is subjected to ∇*T_x_*. (**B** and **C**) The AMTP and the PNE, respectively. The former results when a nonzero *zy* component of magnetization is subjected to ∇*T_y_*. The latter results when an *xy* component of magnetization is subjected to ∇*T_x_* and is maximum when the two are collinear. The AMTP and PNE lead to an electric field generated along *y*, *E_y_*.

∇***T*** describes the thermal gradient in the *x*, *y*, and *z* directions. The terms in the Seebeck tensor *S* are the thermopowers that result from the various effects considered for our geometry, which can be seen in Fig. 1 and discussed below. The individual TE effects that result in a longitudinal voltage along *y* and a wire of width *w*(*x*) are presented in Table 1, where the TE voltages at the tip location (***x***_**0**_ and ***y***_**0**_) are defined.

**Table 1.** Overview of the TE terms in Eq. 1, detailing their geometrical arrangement for the geometry imposed in this work and the expression that led to a longitudinal electrical field along *y.*

Thermoelectric effect (μV)	Geometrical configuration (*E*_*y*_≠0)	Expression
*V*_ANE_(*x*_0_, *y*_0_)	ANE coefficient {$\hat{m}\times \;\nabla T$}	$\int_{x}\frac{\mathrm{dx}}{w(x)}\int_{y}\left[S_{N}\frac{m_{z}}{|m|}\right]\;\nabla T_{x}(x_{\;0},y_{\;0})\mathrm{dy}$ (2)
*V*_PNE_(*x*_0_, *y*_0_)	Seebeck coefficient {∇***T*** ⊥ $\hat{m}$} (PNE)	$\int_{x}\frac{\mathrm{dx}}{w(x)}\int_{y}\left[(S_{∥}-S_{\;⊥})\frac{m_{x}m_{y}}{{|m|}^{\;2}}\right]\;\nabla T_{x}(x_{\;0},y_{\;0})\mathrm{dy}$ (3)
*V*_AMTP_(*x*_0_, *y*_0_)	Seebeck coefficient {∇***T*** ∥ $\hat{m}$} (AMTP)	$\int_{x}\frac{\mathrm{dx}}{w(x)}\int_{y}\left[S_{\;⊥}\left(\;1-\frac{{m_{y}}^{\;2}}{{|m|}^{\;2}}\right)+S_{∥}\frac{{m_{y}}^{\;2}}{{|m|}^{\;2}}\right]\;\nabla T_{y}(x_{\;0},y_{\;0})\mathrm{dy}\;$ (4)

We assume no TE contributions generated from a thermal gradient along the *z* axis, ∇*T_z_*, as any contributions would be negligible due to the 0.6 nm thickness of the CoFeB wire relative to its lateral dimensions (*8*, *9*).The ANE describes the formation of an electric field due to the transverse scattering of thermally generated spin-polarized charge carriers in a magnetic material analogous to the anomalous Hall effect. The microscopic origin of the effect results from a combination of intrinsic and extrinsic phenomena (*37*) including topologically induced Berry curvature (*38*), side jump (*39*), and skew scattering (*40*). The geometrical arrangement of the ANE in a wire with perpendicular magnetic anisotropy (PMA) is schematically shown in Fig. 1A, where the cross product of *m_z_* and thermal gradient along the *x* axis, *T_x_*, leads to a transverse component to the electric field along y, ${\overset{⇀}{E}}_{y}$.

The expression for the ANE is presented in Table 1, where the coefficient, *S_N_*, is the ANE Seebeck thermopower (μV K^−1^), and is related to the ANE coefficient, *N*_ANE_, by $S_{N}(\mu_{0}M_{s}{)}^{-1}$ (*17*, *22*), where μ_0_ is the vacuum permeability and *M_s_* is the saturation magnetization. We also consider TE contributions that arise when the magnetization is coplanar to the local heat current, as is the case when a domain wall is present. This results in TE contributions from the AMTP and the PNE depending on the spin contribution in the coordinate system. The geometrical arrangements of nonzero coplanar TE contributions are schematically shown in Fig. 1 (B and C). Here, a nonzero spin-orbit coupling (***L*** ∙ ***S*** ≠ 0) results in spin mixing and spin flip *sd* scattering of majority conduction *s* electrons into minority hole states in the *d* band, increasing the resistance (*41*). The underlying orbital anisotropy of the empty *d* band electronic states leads to a variation in the scattering cross section when the current is parallel or perpendicular to the local magnetization direction, conserving momentum. Therefore, the TE response is highly sensitive to small changes in the magnetization direction as a result of cross-sectional scattering. This anisotropy is introduced by nonequal Seebeck coefficients, *S*_∥_ and *S*_⊥_, which describe the longitudinal and transverse TE effects, respectively, where a second rank tensor is used to understand their relationship. We derive the equations for TE response shown in Table 1 in section S1.

## RESULTS

### Experimental principle of sThEM

**Fig. 2.**
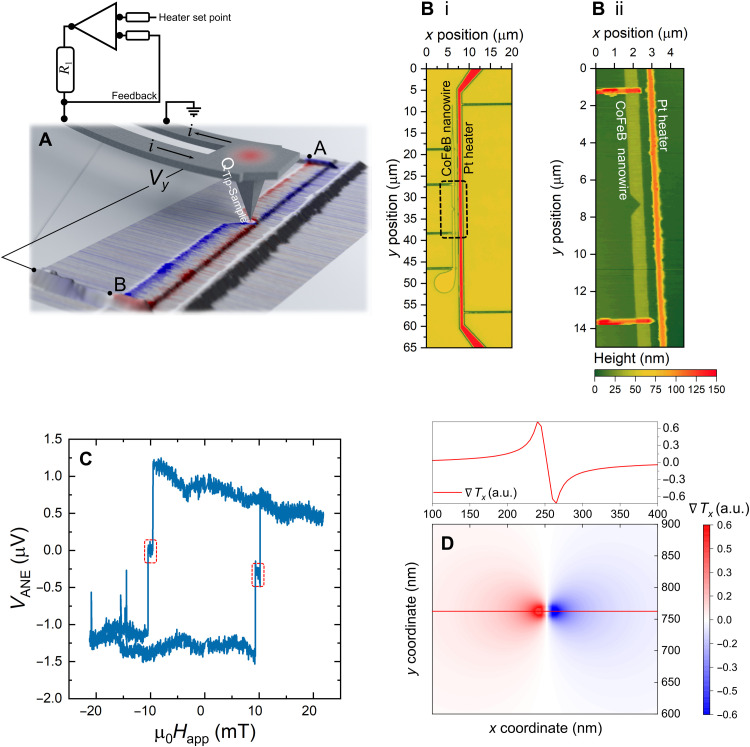
Local manipulation of nonequilibrium spin currents. (**A**) Schematic of the sThEM measurement setup showing the heated SThM probe used to apply the local heat current and thermal gradient. A voltage measurement is made along the device (*y*) and the integrated response is mapped pixelwise. (**B**) Confocal micrograph (i) and atomic force micrograph (ii) highlighting the main device features and the notched region of the magnetic nanowire. (**C**) Thermoelectric magnetotransport plot showing the magnetization reversal process for the magnetic nanowire; domain wall pinning fields are indicated by the red dashed lines. (**D**) Calculated thermal gradient [in arbitrary units (a.u.)] along *x* (∇*T_x_*) when the probe is positioned at the center of the CoFeB wire; inset plots the horizontal profile along the center point.

The key features of the sThEM technique are shown in Fig. 2A. The measurement is performed with a scanning thermal microscopy (SThM) (*42*) probe to provide a localized heat current. The probe is Joule-heated by the application of a voltage to a microfabricated resistor that forms the cantilever, proximal to the Si probe apex (*43*), heating the sample. The nanoscale dimensions of the heated probe provide a highly localized heat source to the sample at the tip location. By raster scanning the heated probe over the magnetic structure, we can sample the local TE response that is generated, thus mapping the TE response in two dimensions to high precision.

Probing the spin texture of a domain wall through its TE response requires the ability to controllably pin a domain wall with a specific and predictable spin configuration. To achieve domain wall pinning in our experiments, we introduce a notch in our ultrathin Pt/Co_60_Fe_20_B_20_/Pt trilayer-based device (*28*) (see Methods). Figure 2B (i and ii) shows a confocal and an atomic force micrograph, respectively, highlighting the device topology. The Pt heater is used to characterize the global TE response of the device by magnetotransport (see Methods). A global ANE measurement is shown in Fig. 2C, where the applied magnetic field is applied along the *z* axis, out of the sample plane. Domain wall pinning is indicated by the step, red dashed lines, in the TE transport data. The magnetotransport demonstrates that a strong PMA is present in our devices.

To correlate the sThEM and modeling results in the following sections, the thermal gradient generated by the heated probe was calculated by solving the three-dimensional Poisson equation (see Methods). Figure 2D shows the calculated *x* component of the local thermal gradient ∇*T_x_*, at the wire center. A cross-sectional line profile is extracted along the path of maximum ∇*T* to demonstrate the subsequent inversion of thermal gradient and hence direction of the spin-polarized current at either side of the heated-probe apex (inset in Fig. 2D). In this work, we assume the maximum tip temperature to be *T*_tip_ ≈ 330 K; further details are shown in section S2.

### Local sThEM-saturated state and trapped Néel domain wall

**Fig. 3.**
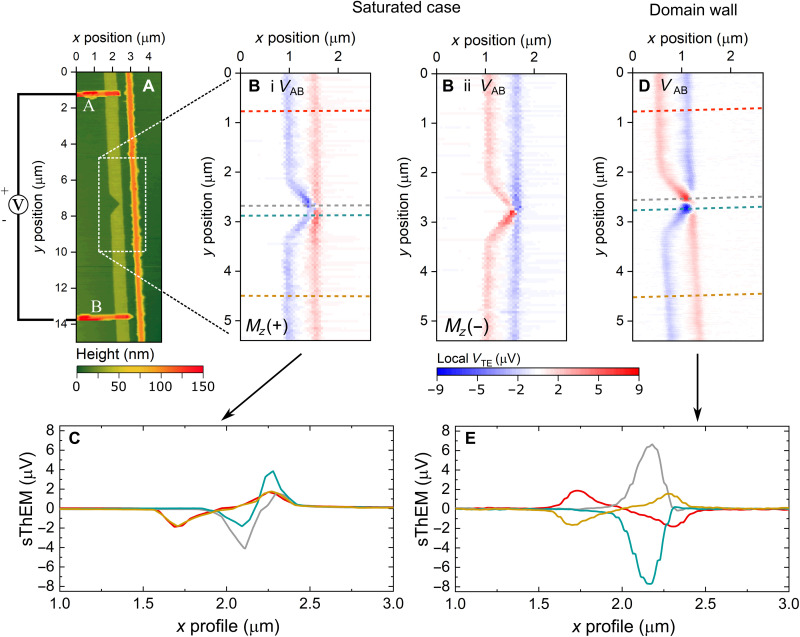
Local TE response from a pinned Néel wall. (**A**) Atomic force micrograph of the nanowire region of interest. (**B**, i and ii) Local sThEMs for two magnetization states, *m_z_* = ± 1, respectively. (**C**) Line profiles taken across the data in the saturated state (Bi). (**D**) sThEM micrograph for the pinned domain wall. (**E**) Line profiles taken in equivalent regions to (C) for the domain wall state (D). Dashed lines are representative of the locations where the profiles (C) and (E) were taken. [Line profile abscissa in (C) and (E) are transformed from the micrograph axes, (B) and (D), for clarity and to center features of interest for comparison purposes.]

Figure 3A displays a labeled atomic force micrograph of the device, where electrical contact points, *A* and *B*, were used to record the local TE response. Figure 3B (i and ii) shows sThEM micrographs of the CoFeB nanowire at remanence, in both positive and negative magnetization orientations along the *z* axis. A voltage maximum/minimum is established along the long edges of the device as indicated by the blue and red regions. The signal reverses sign between Fig. 3B (i and ii) by applying a reversed magnetic field, which confirms its magnetic origin.

It is possible to intuitively explain the sThEM profile in Fig. 3B by considering the earlier magnetotransport measurements and thermal modeling from Fig. 2 (C and D, respectively). The square shape of the magnetization reversal curve (Fig. 2C) indicates that ∣*M_z_*∣/*M_s_* ≈ 1 at remanence. Therefore, the thermal gradient generated along the short axis,∇*T_x_*, generates a transverse electric field, *E_y_*. In this configuration and geometry, the manifold of TE phenomena is reduced solely to the ANE (Table 1). As the probe traverses the nanowire, the signal inverts, which can be understood from the integrated response of ∇*T_x_* across the nanowire (Fig. 2D).

Cross-sectional profiles of the saturated state are presented in Fig. 3C, which were taken at the positions of maximum signal at the notch center and far from the notch. We observe an increase in the maximum signal at the notch center, which arises due to the reduced geometrical symmetry of the device in this region. This subsequently results in an asymmetry of the local thermal gradient along *y*, presented in section S2. The increased TE response is observed by the difference in the amplitude of the profiles taken both at the notch center and far from the notch, respectively. A nonzero integrated ∇*T_y_* leads to an additional AMTP contribution to the TE voltage due to the asymmetric device geometry imposed by the presence of the notch. Further insight into this is presented in the modeling section. We also note that variations of the signal response at the notch site could result from a combination of slight variations in the physical device geometry combined with asymmetries in the tip-sample contact, which would affect the thermal heat flux to the sample spatially.

The presence of the domain wall gives rise to a reversal in the signal of the TE response away from the notch depending on the local magnetization direction (Fig. 3D). This is consistent with the magnetotransport results, where the longitudinal electric field *E_y_* results from the ANE because of a nonzero *z* component of the magnetization. Above the notch center, the data presented in Fig. 3D match the signal direction and magnitude seen in Fig. 3B (i and ii); this is further demonstrated by the line profiles presented in Fig. 3 (C and E).

However, we now measure a large TE response of $V_{\mathrm{TE}}^{\mathrm{Notch}}$ ≈7 to 8 μV at the notch center, which is greater than that of the saturated state, $V_{\mathrm{TE}}^{\mathrm{Sat}}$ ≈4 μV (Fig. 3C). This is best demonstrated in the cross-sectional profiles taken for both the saturated and domain-wall case (Fig. 3, C and E), where we plot the two datasets adjacently with identical scaling.

We propose that the increased TE response arises from nonzero components of the magnetization in the *zy* plane, which arises from the presence of a Néel domain wall. In-plane components of the local magnetization give rise to contributions from the AMTP and the PNE because of the components in the *zy* plane and/or the *xy* plane, respectively, as detailed in Table 1. Inspection of Eq. 1 leads to the supposition that the domain wall itself leads to an additional TE response and a noticeable impact on the total TE signal.

## DISCUSSION

To unambiguously determine the additional TE response from the pinned Néel domain wall, we have developed a full TE model. We combine temperature-dependent micromagnetic modeling using an LLB micromagnetic framework (*32*–*34*) and thermal modeling of the heated-probe nanowire system using finite element modeling (FEM).

### Calculated TE response from ideal Bloch and Néel domain walls

**Fig. 4.**
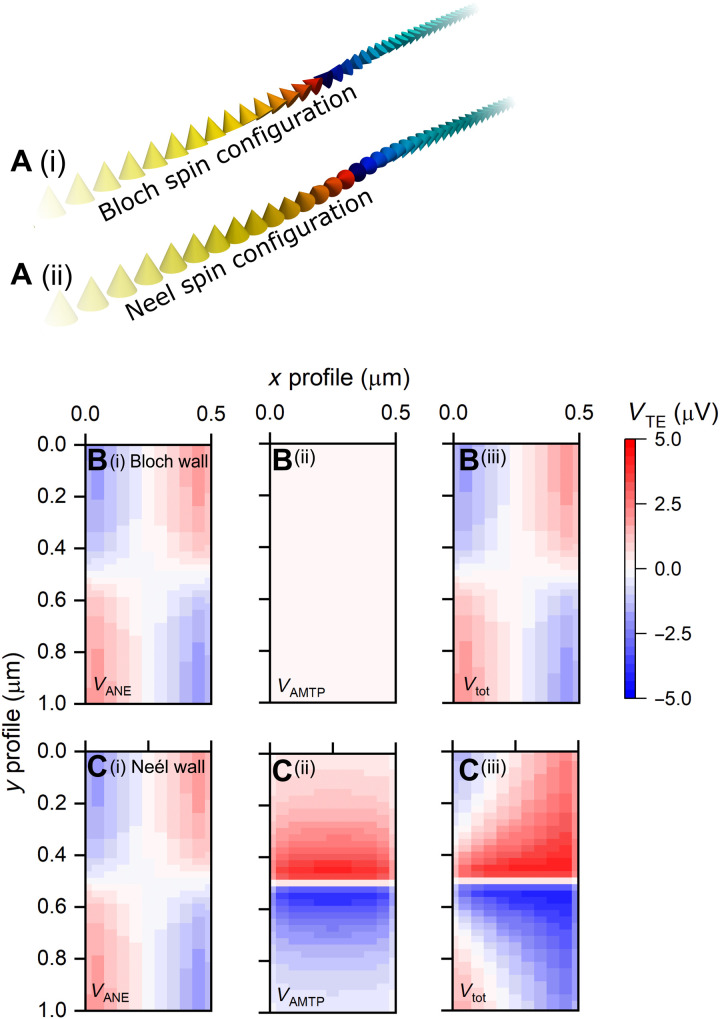
Local TE response from ideal Bloch- or Néel-type domain wall. (**A**) Cross-sectional vector fields used in the TE calculations, showing the *m_z_* components for the Bloch and Néel domain wall configurations (i and ii, respectively). (**B**) Calculated individual TE responses for the Bloch domain wall; *V*_ANE_, *V*_AMTP_, and *V*_tot_ (i to iii, respectively). (**C**) Calculated individual TE responses of the Néel domain wall; *V*_ANE_, *V*_AMTP_, and *V*_tot_ (i to iii, respectively). *V*_tot_ map (Ciii) contains the additional response from the *V*_AMTP_, allowing distinguishable wall types.

To gain an intuitive understanding of the role of individual TE contributions, we start with analytically described Bloch- and Néel-type walls in a straight wire with no notch, neglecting thermal effects on the magnetization dynamics. Figure 4A (i and ii) shows schematics of the spin vector fields for the ideal Bloch and Néel walls used in the TE calculation. The domain wall width was fixed at 20 nm, and the full width at half maximum of the heat spot was 100 nm.

Figure 4 (B and C) shows the individual TE responses resulting from the ANE, AMTP, PNE, and total voltage for the Bloch- and Néel-type spin configurations, respectively. In this system, we assume a set of Seebeck parameters, where *S*_∥_***>****S*_**⊥**_, in line with the fitted parameters that were extracted from the experimental data (see section S3).

Figure 4Bi shows that the local ANE response exhibits the expected behavior and replicates the experimental observations of the saturated regions above and below the domain wall (Fig. 3D). Above and below the Bloch domain wall, the ANE response inverts in sign due to the changed sign of *m_z_*. In the domain wall, the ANE contribution diminishes as *m_z_* → 0 (Eq. 2) Figure 4Bii reveals that no AMTP response arises in our measurement geometry. Because of the symmetric decay of the *y* component of the thermal gradient at the tip position, the *S*_⊥_term above and below the probe is fully canceled. As the Bloch wall is confined to the *xz* plane, no AMTP or PNE response is expected in our measurement geometry (Eqs. 3 and 4, respectively). Therefore, the total TE response for the Bloch domain wall is purely due to the ANE (Fig. 4Biii).

Next, we consider the case for the Néel spin configuration. The TE contribution from the ANE (Fig. 4Ci) is identical to the Bloch wall case, as the *m*_z_ components are identical for both models. However, in the domain wall, we obtain a large TE response from the *V*_AMTP_ (Fig. 4Cii), which results from the rotation of the spins in the *yz* plane. The total TE response from the Néel domain wall, displayed in Fig. 4Ciii, is now entirely distinct from the previous Bloch wall case. For both the ideal Bloch and Néel domain walls, there is no rotation of spins in the *xy* plane; therefore, *V*_PNE_ is zero for both scenarios.

Therefore, from our simple calculation, we demonstrate that it is possible to discriminate between the Bloch and Néel domain wall in our measurement geometry. However, it is not possible to determine the domain wall chirality. This is due to squared term in Eq. 3, which masks the domain wall chirality and leads to a degenerate state for the rotation of spins in the *yz* plane.

### Effect of notch on calculated TE response for ideal Bloch and Néel domain walls

**Fig. 5.**
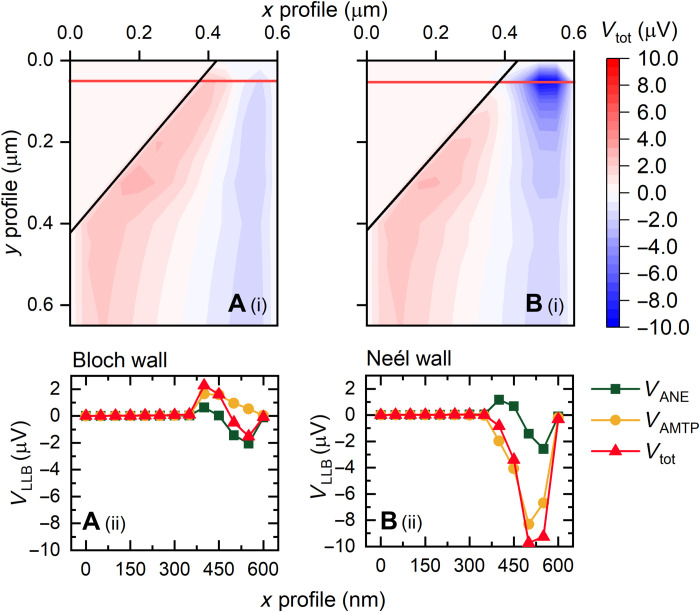
Local TE response from ideal Bloch- or Néel-type domain walls with geometrically induced thermal asymmetry. (**A**) *V*_**tot**_ TE response map for a Bloch wall and line profile taken 50 nm below the notch center (i and ii, respectively); *V*_AN*E*_, *V*_AMTP_,and *V*_tot_ represented by green squares, yellow circles, and red triangles, respectively. (**B**) *V*_tot_ TE response for a Néel wall and line profile taken 50 nm below the notch center (i and ii, respectively). Profile locations are indicated by the red solid line in (Ai) and (Bi). A large response in *V*_AMTP_ dominates *V*_tot_ in (Bi) due to the Néel wall.

Now that the magnetic origins to the TE signals are understood, we can build the geometric asymmetry of the notch into the analytical analysis. Figure 5 shows the calculated TE response for Bloch and Néel domain walls located at the notch position. Both domain walls are defined by an analytical profile, with width ≈15 nm. Here, we use the extracted Seebeck coefficients detailed in section S3, *S*_∥_ = −2.0 μV K^−1^ and *S*_⊥_ = −0.079 μV K^−1^.

Figure 5Ai exhibits the total TE response, *V*_tot_, from the wire when the Bloch wall is positioned at the notch center. Horizontal line profiles for *V*_tot_ were extracted at *y* = 50 nm and plotted in Fig. 5Aii alongside the ANE and AMTP voltage components. *V*_ANE_ shows the same expected response as described for Fig. 4. However, *V*_AMTP_ generates an additional contribution to the total TE voltage due to the geometrically induced thermal asymmetry discussed in the previous section. The signal along the notch at the left-hand edge of the device is enhanced as this is where the thermal asymmetry is the largest. We also observe this geometrically induced signal in the experimental data, which is most prominent in the saturated states (Fig. 3B, i and ii), where the signal at the notch is enhanced.

Figure 5Bi displays *V*_tot_ for the case where the Néel wall is located at the notch center, which has a very different response to Fig. 5Ai. The horizontal line profiles in Fig. 5Bii show a comparable *V*_ANE_ contribution to the Bloch wall. However, a far more significant contribution to the total TE voltage arises from the *y* component of magnetization and an enhancement of *V*_AMTP_, complementing the results for the straight wire in Fig. 4. The enhanced response modifies *V*_tot_ such that the contribution from the Néel wall is the dominant term of the TE response at the notch location. The thermal asymmetry–induced TE response is also observed; however, because of the large signal from the *y* component of the magnetization, it is comparatively small. This strongly agrees with the experimental data presented in Fig. 3 (B to E).

The role of thermal asymmetry is explored even further in section S4, where the separate voltage contributions of *S*_⊥_ and *S*_∥_ are analyzed. The TE response of the Bloch wall is independent of the spins in the domain wall (Eq. 4). In this case, a TE response only arises due to thermal gradient asymmetry, *S*_⊥_. As such, the TE response is identical to the saturated wire case away from the notch, and we find no signal contribution from *S*_∥_. The TE response for the Neel wall due to the *S*_⊥_ term is identical to the Bloch wall case. However, a sizable TE response from *S*_∥_ arises due to the rotation of spins in the *yz* plane, as shown in Eq. 4, which explains the dominant contribution to the total signal in the Néel wall. It is from this geometric argument that we can distinguish the TE response for the two domain wall configurations considered in this work.

### Calculated TE response from relaxed Néel domain wall: Full-scale micromagnetic modeling based on the LLB equation

As the measurement approach uses a heated probe, we hypothesize that the induced thermal gradient could drive spin-polarized electrons out of equilibrium leading to a net flow of angular momentum. As such, we expect that a magnonic coupling to the domain wall could also arise due to the presence of these spin currents. Angular momentum exchange can result from a thermally generated spin transfer or spin orbit torques (*21*, *44*) or due to heat flow carried by thermally excited magnons (*45*–*47*) (magnonic spin torques) and can lead to effective coupling with magnetization textures.

Therefore, we have used micromagnetic modeling within the LLB framework combined with the temperature output from FEM for the heated probe-sample system (see Methods and section S5). This allows us to gain an intuitive understanding of the role of temperature gradients at micromagnetic time scales. Here, we explicitly consider torques induced by heat flow carried by magnonic spin currents. In this microscopic picture, the magnon density increases at the probe position, where the temperature is highest, and diffuses into cooler regions. Close to the domain wall, the magnon flow leads to an exchange of angular momentum, causing the domain wall to displace toward the hotter region (*47*).

**Fig. 6.**
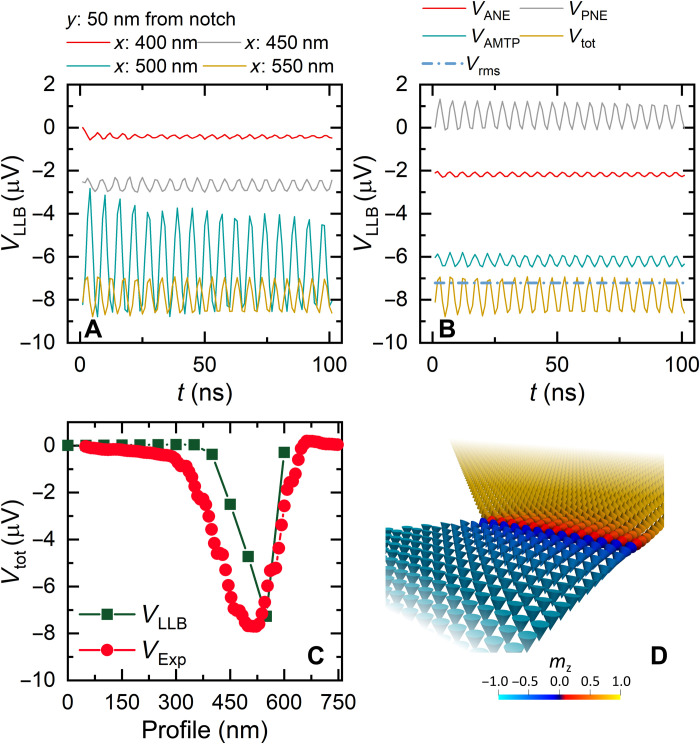
LLB simulations showing dynamic TE response due to domain wall oscillations, *T_c_*=580 K. (A) Temporal plot of *V*_tot_ at *x* = 400,450,500, and 550 nm and *y* = − 50 nm from the notch center, displaying the oscillatory voltage response. (**B**) Temporal plot of the individual voltage components *V*_ANE_, *V*_PNE_, *V*_AMTP_, *V*_tot_, and *V*_RMS_ at *x* = 550 nm and *y* = − 50 nm. To extract a unique value for the *V*_LLB_, the root mean square voltage, *V*_RMS_, was calculated. (**C**) Line profile (*x* direction) comparison of the LLB modeling and experimental data, *V*_LLB_ and *V*_Exp_, respectively. (**D**) Spin vector field for the relaxed Néel domain wall *K*_ani_ = 8 × 10^5^ J m^−3^, and *T_c_* = 580 K. The heat flux was kept constant throughout all simulations.

To investigate the role of the thermal contribution to the magnetization dynamics, we have evaluated the temporal response of an energetically relaxed Néel domain wall. Figure 6A shows the temporal evolution of the calculated *V*_tot_ for different probe positions along *x* for a duration of 100 ns. *V*_tot_ displays an oscillatory output due to the cyclic attraction of the domain wall against the thermal gradient, followed by destabilization and relaxation back into the notched pinning site. The amplitude of the oscillatory response is maximum when the probe is positioned at the center of the microwire commensurate with the largest overlap of ∇*T_y_* and the domain wall. This oscillatory behavior is explained by the presence of a net flow of magnonic current uniformly about the tip position. These currents initially interact with the domain wall by an exchange of angular momentum and pull it in the direction opposite to the magnon flow. When the wall is displaced from the equilibrium position, the attractive nature of the notch combined with opposing magnonic currents subsequently push the domain wall back into the starting position at the notch center. We hypothesize that localized heat gradients could be further exploited to drive or measure dynamic behavior for localized spin wave and localized magnonic applications. The efficiency of this process is dependent on the location of the probe along *x*, which we conclude from the magnitude of the oscillations.

To understand the individual contributions to the total TE signal, we plot the time evolution of the separate components in Fig. 6B, where we find that the separate TE contributions also oscillate. We note a nominal contribution arising from *V*_PNE_ (Eq. 3), due to a nonzero magnetization component lying in the *xy* plane. However, because the prominent spin component is still aligned along *y*, the *V*_AMTP_ contribution remains the dominant voltage term in the domain wall, consistent with the conclusions drawn from the previous sections.

To compare the modeled TE response from the dynamic system to the quasi-static sampling rate of the experimental technique (order of milliseconds), the root mean square value (*V*_RMS_) of the modeled data is calculated as a function of tip position. Figure 6C shows a comparison of the line profiles of the TE output from the calculation and the experimental study. We find a strong quantitative agreement between the two, indicating that the experimental TE response arises owing to the Néel nature of the pinned domain wall. The spin vector field for the simulated domain wall is plotted in Fig. 6D, where the *z* component is represented by the color scale. The minimized state can be described predominantly as a Néel domain wall. This is to be expected for a PMA thin film of this thickness due to spin reorientation in the *xy* plane, which minimizes the magnetostatic energy from the planar surfaces at the cost of increased domain wall width (*48*, *49*).

In conclusion, we have conducted a comprehensive investigation of local TE effects from a single domain wall by scanning TE microscopy. By driving a nanoscopic heat current from a heated SThM tip in thermal contact with the sample, we excite and map a highly localized TE response. This laboratory-based approach offers an unprecedented insight into TE phenomena at the nanoscale. By isolating the individual TE contributions, it is now possible to distinguish the plane of rotation of the spins within a domain wall and discern domain wall properties, such as its Néel-like character. This now allows the unambiguous characterization of Bloch or Néel domain walls using nanoscopic heat sources to drive a TE response. Highly localized TE mapping will open new avenues for rapid characterization of novel TE devices and phenomena at the nanoscale, accelerating magnetic energy harvesting technologies and thermally driven spintronics. Our micromagnetic modeling results also indicate that nanoscopic heat sources could potentially be used to excite magnetization dynamics in confined systems for spin wave and magnonic applications. As such, this a key advancement in exploring the limits of TE material systems at fundamental length scales, as shown here for domain walls.

## METHODS

### Thin-film growth and device fabrication

The perpendicular magnetic anisotropy trilayer architecture is composed of Ta(4 nm)/Pt(3 nm)/Co_60_Fe_20_B_20_(0.6 nm)/Pt(3 nm), which was sputtered onto a Si/SiO_2_ substrate. The nanowire was patterned from the continuous CoFeB thin-film trilayer by electron beam lithography in combination with argon ion etching. In a second lithography step, the contacts and the heater were fabricated in a lift-off process using sputter deposition. Before the deposition of the contacts and heater, the area was cleaned in situ by low-energy argon ions to ensure good electrical contact. The deposited contacts and heater consist of a 5 nm Ta adhesion layer and a 95 nm Pt layer of 95 nm thickness.

### Magnetothermoelectric transport

Global TE transport measurements were performed outside of the scanning probe microscope in a dedicated experimental setup. The device was electrically contacted to a sample stage, which allows us to monitor the TE response of the CoFeB nanowire and power the Pt heater using a Keysight 34420a nanovoltmeter and a Keithley 2400 current source, respectively. To sweep the magnetic field, we used a GMW 5403 electromagnet, and data acquisition was performed by the bespoke LabVIEW software “Modulab.”

### Scanning thermoelectric microscopy

Measurements were performed by an Anasys NanoIR2 scanning probe microscope with an Anasys AN-200 thermal probe that provided the localized heat current. All measurements were performed under ambient conditions using “contact mode” atomic force microscopy with an applied set point force of ~14 nN. The probe was raster-scanned over the sample area, and the TE response from the CoFeB nanowire was measured pixel by pixel with a Keysight 34420a nanovoltmeter with an integration time of 40 ms, which was fed into the atomic force microscope through an auxiliary input. The probe heating power was kept constant using active feedback to a potential divider circuit. Data processing was performed to remove any background offset to zero when the probe was not in contact with the device (i.e., zero TE response).

### Domain wall pinning protocol

To set the magnetic states of the device during sThEM, we used a permanent magnet, which was applied along *z*, ex situ from the scanning probe microscope. To repeatably pin the domain wall for sThEM measurement, the sample was loaded into the magnetotransport setup described above at an angle of approximately 19° from the surface normal to break the coordinate system symmetry and promote domain wall pinning. The field was swept while the TE response was monitored by the method described above. The sequence was stopped abruptly when plateau was observed in the TE response indicating domain wall pinning. The applied field was reduced to zero and transferred for subsequent sThEM measurements.

### Micromagnetic modeling and TE calculation

Micromagnetic modeling has been performed using a homemade program based on the LLB equation allowing variable magnetization length (magnitude of the magnetization vector, normalized to its magnitude at *T* = 0 K) along the heat gradient. The following zero-temperature parameters were assumed: saturation magnetization (*17*) *M*_*s*_(*T* = 0 K) = 1.03 × 10^6^ A m^−1^, the exchange constant *A*_ex_ (*T* = 0 K) = 1.2 × 10^−11^ J m^−1^, and the perpendicular anisotropy *K*_*u*_ (*T* = 0 K) = 0.90 × 10^6^ J m^−3^ giving the domain wall width ≈ 15 nm at 300 K, close to the experimental value. The temperature dependence of magnetization was taken from the Langevin function with the Curie temperature *T_c_* = 580 K, and the scaling relations with reduced equilibrium magnetization $\$m(T)=\frac{M_{s}(T)}{M_{s}(0\;K)}\$$ were assumed for the temperature dependence of anisotropy (*50*) *K*(*T*) = *K*(0) × *m*(*T*)^3^ and exchange stiffness (*51*) *A*(*T*) = *A*(0) × *m*(*T*)^1.76^. The TE parameters were fitted to the measured voltage in the saturated case (see section S3). We discretize a strip of dimensions 1500 nm × 500 nm × 1 nm into 5 nm × 5 nm × 1 nm voxels. The V-notch dimensions were such that the vertical notch height (base of the effective triangle removed from the microstrip) was 870 nm, and the notch width (minimum width of the device at the notch) was ≈ 50 nm.

### Thermal modeling

Finite difference modeling was used to calculate the head gradients in the probe-sample volume by solving the Poisson equation using the successive overrelaxation method (*52*). For each probe position on the sample, an individual calculation was performed, including building a mesh and reaching convergence in the heat flux through the probe-sample system.

## Supplementary Materials

### This PDF file includes:

Supplementary Text Figs. S1 to S10 Tables S1 and S2 References
