# Supplementary material for: Local thermoelectric response from a single Néel domain wall
Source: Sci Adv. 2022 Nov 23;8(47):eadc9798. doi: 10.1126/sciadv.adc9798 (PMC9683730; doi:10.1126/sciadv.adc9798)
Supplement: Supplementary file 1 — Supplementary Text Figs. S1 to S10 Tables S1 and S2 References [file sciadv.adc9798_sm.pdf]

Science Advances
AAAS

Supplementary Materials for

Local thermoelectric response from a single Néel domain wall

Robert Puttock *et al.*

Corresponding author: Robert Puttock, robb.puttock@npl.co.uk; Craig Barton, craig.barton@npl.co.uk

Sci. Adv. **8**, eadc9798 (2022)
DOI: 10.1126/sciadv.adc9798

This PDF file includes:

Supplementary Text
Figs. S1 to S10
Tables S1 and S2
References

Supplementary Text

1. Derivations of the thermoelectric expressions

We start by assuming the magnetisation aligned along z-direction, perpendicular to the sample/substrate surface. The thermal probe that is used to apply the local thermal gradient induces an electric field pointing away from the heat source at the probe apex. For out-of-plane uniform magnetisation, the induced voltage is described by the Seebeck tensor written as following:

$$\nabla V = -\begin{pmatrix} S_\perp & -S_N & 0 \\ S_N & S_\perp & 0 \\ 0 & 0 & S_\parallel \end{pmatrix} \nabla T \quad (1)$$

where $S_N$is the anomalous Nernst coefficient, $S_\perp$and $S_\parallel$ are the transversal and longitudinal Seebeck coefficients, respectively and ∇T is the temperature gradient.

For an arbitrary magnetisation direction presented in Fig. 1 in the main text, the coordinate system should be rotated the way the magnetisation direction turns parallel to the z-axis and the transport matrix S' becomes

$$S' = R_{zy}(\varphi, \theta) S_z R_{zy}^{-1}(\varphi, \theta) \quad (2)$$

$$= \begin{pmatrix} \cos\theta\cos\varphi & -\sin\varphi & \cos\varphi\sin\theta \\ \sin\varphi\cos\theta & \cos\varphi & \sin\theta\sin\varphi \\ -\sin\theta & 0 & \cos\theta \end{pmatrix} \begin{pmatrix} S_\perp & -S_N & 0 \\ S_N & S_\perp & 0 \\ 0 & 0 & S_\parallel \end{pmatrix} \begin{pmatrix} \cos\theta\cos\varphi & \sin\varphi\cos\theta & -\sin\theta \\ -\sin\varphi & \cos\varphi & 0 \\ \cos\varphi\sin\theta & \sin\theta\sin\varphi & \cos\theta \end{pmatrix} \quad (3)$$

Here θ and φ are magnetisation vector polar and azimuthal angles, respectively and R is the rotational matrix whose indices indicate the rotational plane. As the voltage signal is measured in y-direction, and the thermal gradient in z-direction is neglected because of the negligible thickness of the ferromagnetic layer, we only need the yx and yz terms of the transport matrix (for magnetisation in the yx and yz plane) to describe the voltage signal:

$$S'_{,(yx)} = S_N \cos\theta + \frac{1}{2}(S_\parallel - S_\perp)\sin^2\theta\sin(2\varphi) \quad (4)$$

$$S'_{,(yz)} = S_\perp(1 - \sin^2\varphi\sin^2\theta) + S_\parallel \sin^2\theta\sin^2\varphi \quad (5)$$

Or in cartesian coordinates

$$S'_{,(yx)} = S_N \frac{m_z}{|m|} + (S_\parallel - S_\perp)\frac{m_x m_y}{|m|^2} \quad (6)$$

$$S'_{,(yz)} = S_\perp\left(1 - \frac{m_y^2}{|m|^2}\right) + S_\parallel \frac{m_y^2}{|m|^2} \quad (7)$$

Convoluting the above expressions with thermal gradients following (1) and integrating the voltage across the stripe we obtain the total voltage as a sum of effects coming from gradients in two different directions and comprising the longitudinal and planar transport effects.

$$V_{yx} = -\int_{x_{min}}^{x_{max}} \frac{dx}{w(x)} \int_{-L/2}^{L/2} \left[S_N \frac{m_z}{|m|} + (S_\parallel - S_\perp) \frac{m_x m_y}{|m|^2} \right] \nabla_x T(x, y, x_0, y_0) dy \quad (8)$$

$$V_{yz} = -\int_{x_{min}}^{x_{max}} \frac{dx}{w(x)} \int_{-\frac{L}{2}}^{\frac{L}{2}} \left[S_\perp \left(1 - \frac{m_y^2}{|m|^2}\right) + S_\parallel \frac{m_y^2}{|m|^2} \right] \nabla_y T(x, y, x_0, y_0) dy \quad (9)$$

where *(x_0, y_0)* define the position of the heat spot, $w(x)$ is the stripe short dimension (variable because of the notch presence between x_{min} and x_{max}) and L is the stripe length.

2. Considerations of the thermal gradient from the probe and notch asymmetry.

To demonstrate the role of the notch on the thermal asymmetry, we present examples of cases below. Firstly, Fig. S1(a) shows a heatmap with the tip in the centre of the nanowire but away from the notch. Fig. S1 (b) and (c) shows the thermal profile and gradient along the x-axis, respectively. We see that the thermal gradient is relatively symmetric radially from the tip position.
Figs. S2 (a-c) are heatmaps for three different heat spot positions close to the constrained notch. Figs. S2 (d-f) show the thermal distribution along x-direction and across the heat spot maximum. Figs. S2 (g-i) show the thermal gradient along x-direction along the same line. The data present thermal asymmetry due to the notch influence.

Fig. S3 (a-b) are spatial representations of the thermal gradients along x when the probe is positioned near the wire edge. The data show that when the probe is close to the notch, Fig. S3 (a), the thermal asymmetry either side of the tip position is greater than when the probe is moved further away, Fig. S3 (b).

Fig. S3 (c-d), show the y thermal gradients at the same locations as in (a-b), respectively. Again, the notch edge introduces thermal asymmetry, this time along the y direction. These thermal gradient maps assist our understanding when the thermoelectric effects are considered in the main text, Fig. 5 and below, Fig. S9 and S10. These variations in the thermal gradients combined with our analytical model, Eqn. 1- 9, help to explain why there is an increase in the signal in the notch region when the wire magnetisation is saturated (i.e. without domain wall).

Fig. S3 (e-f), show the local magnetisation modulus (normalised to magnetisation saturation at T=0K) due to the presence of the probe, as calculated by the LLB modelling. This data shows that, in addition to the thermal gradient asymmetry in the notch, the geometry of the notch also affects the local reduction in the magnetisation. This means that there are two contributions to the enhanced notch signal in the wire: purely thermal and magnetothermoelectric. The consequences of these contributions on the domain wall stability are explored further in Section 5 of the SI.

3. Estimation of the Seebeck parameter from the experimental data

To input accurate Seebeck coefficients into our thermoelectric calculations it is necessary to extract them by fitting the response from the experimental data. Since the heat spot is localised, far from its position, the voltage is zero. Moreover, far from the notch, the signal is the same as in a stripe without a notch. For a homogeneously magnetised sample, the voltage density (ΔV, normalised to the $\mu_0 M_s$ factor) has the following form (see Eqs.(6-7)):

$$\Delta V = -\left(S_N \frac{m_z}{|m|} + \frac{m_x m_y}{|m|^2}\right) \nabla_x T - \left[S_\perp \left(1 - \frac{m_y^2}{|m|^2}\right) + S_\parallel \frac{m_y^2}{|m|^2}\right] \nabla_y T \tag{10}$$

where m_i is the magnetisation along the stated coordinate direction and $\Delta_n T$ is the temperature gradient, S_N is the Nernst coefficient, and $S_\perp$ and $S_\parallel$ are the perpendicular and parallel Seebeck coefficients, respectively.

Let us consider a saturated sample in +*Z* and -*Z* directions, then far from the notch we have

$$\Delta V^{+Z} = -S_N \nabla_x T - S_\perp \nabla_y T \tag{11}$$

$$\Delta V^{-Z} = S_N \nabla_x T - S_\perp \nabla_y T \tag{12}$$

The contribution from the $S_\perp$ part cancels after the integration over the whole sample due to the asymmetric nature of the gradient in y direction from the probe location. Then, the contribution of the anomalous Nernst effect can be obtained from the experimental measurements from a difference $V^{+Z} - V^{-Z}$.

Moreover, let us consider a simplified Gaussian-like heat spot profile with spherical symmetry

$$T(x, y) = T_{max} \exp\left[-\frac{(x-x_0)^2}{2\sigma^2} - \frac{(y-y_0)^2}{2\sigma^2}\right] \tag{13}$$

where T_{max} is the maximum temperature and σ is the spot width. Disregarding the temperature dependence of the saturation magnetisation, the voltage profile can be obtained far from the notch for a strip of infinite length in the y-direction and width of L in the x-direction. Therefore, from Eqs.(8-9) the thermoelectric response of a saturated stripe due to ANE is given by:

$$V_{ANE} = \frac{S_N T_{max}}{\sigma^2} \int_{-\frac{L}{2}}^{\frac{L}{2}} \left[(x - x_0) \exp\left(-\frac{(x-x_0)^2}{2\sigma^2}\right)\right] dx \int_{-\infty}^{+\infty} \exp\left[-\frac{y^2}{2\sigma^2}\right] dy \tag{14}$$

Performing the integral, we obtain the expression for voltage in a stripe far from a notch as a function of the heat spot position along the short direction *x:*

$$V = 2\sqrt{2\pi} T_{max} \sigma S_N \exp\left(-\frac{\frac{L^2}{4} + x_0^2}{2\sigma^2}\right) \sinh\left(\frac{L x_0}{2\sigma^2}\right) \tag{15}$$

Eq. 15 shows that the voltage profile far from the notch is proportional to the factor $T_{max} \cdot S_N$ only, which constitutes our main fitting parameter. The corresponding function is antisymmetric with respect to the stripe centre and has a maximum/minimum at the stripe edges. Far from the edges the function is linear as a function of the spot position x_0, following:

$$V = \sqrt{2\pi} S_N L \frac{T_{max}}{\sigma} \exp\left(-\frac{L^2}{8\sigma^2}\right) x_0. \tag{16}$$

Fig. S4 shows the voltage measured in the experiment far from the notch (black) and those obtained by solving the LLB equation using the simulated heat profile (red). Due to a size difference between experimental device and the simulated system, the experimental data ranges from -350nm to 350nm, whereas the simulated data ranges from -250nm to 250nm. The shapes of the simulated curves agree well with the experimental curves and show a linear behaviour in the stripe centre from which the coefficient $T_{max} \cdot S_N$ is extracted. Reasonable temperature values in agreement with our thermal modelling, together with the corresponding values for the anomalous Nernst coefficient are presented in Table S1 below.

Table S1 shows the fitted values of the $S_N \cdot T_{max}$ and $S_\perp \cdot T_{max}$ products. The temperature maximum is calculated from the original thermal data from thermal calculations. The majority of our calculations use the value of coefficients corresponding to $T_{max} = 327\ K$ since it gave the best compromise with our thermal modelling data and the previously reported value of the anomalous Nernst coefficient (from Krzysteczko *et al.*, Ref. [17].

Analogously, we can perform the sum of voltage signal of the sample saturated in ±Z direction, as shown in Fig. S5:

$$\Delta V^{+Z} + \Delta V^{-Z} = -2S_\perp \Delta_y T. \quad (17)$$

$S_\perp$ cannot be extracted from the data obtained far from the notch due to the symmetry of the gradient in y-direction, which yields zero voltage after integration. We take the average of the experimental data close to the notch at 150nm and 200 nm from its centre. The experimental data is fitted to the numerical dataset using the fitting parameters $S_\perp \cdot T_{max}$, see Fig.S5 The obtained fitted data for various T_{max}, are presented in Table S1.

The comparison of the total voltage obtained in the experiment and in simulations with a zero $S_\parallel$ is presented in Fig. S6. It shows that the signal shape is well reproduced by the modelling, albeit the maximum values are smaller in the modelling.

Note that for the saturated case, the voltage is independent from parallel Seebeck coefficient, which was adjusted later for the maximum signal obtained for a domain wall pinned at the notch centre, see Fig. 5 of the main manuscript. The value of $S_\parallel$ used was -2.0 $uV\ K^{-1}$, which is larger in comparison to literature values. One reason for this discrepancy could arises from the fact that we fix the thermal gradient in the multi-parameter fit. For instance, if the thermal gradient is underestimated, this would lead to an overestimation of $S_\parallel$. To circumvent this, improved calibration of the thermal properties of the probe could lead to more informed inputs required for the thermal modelling.

4. Separation of perpendicular and parallel AMTP response for Bloch and Néel domain walls

As the AMTP Seebeck response contains two terms arising from the perpendicular and parallel Seebeck coefficients it is helpful to separate the two contributions and present them independently. We do this below for the case of the Bloch wall and Néel respectively. As a pure modeling experiment, two synthetic domain walls were created by their known analytical profiles with widths $\delta_w = 20\ nm$ (no energy minimisation was performed). These domain walls were assumed to be pinned at the notch. The output voltage was calculated using the

numerical heat profile described in S2 of the SI. For the thermal transport coefficients, we use the valued from Table S1 corresponding to $T_{max} = 327\ K$ and $S_{||}\ =\ -2.0\ \mu V\ K^{-1}$

Bloch wall: Fig. S7 shows the thermoelectric response for the separated AMTP Seebeck components. Fig. S7 (a) i-ii shows the perpendicular and parallel AMTP Seebeck voltage $V_{A\mathrm{MTP}}\ (\perp)$ and $V_{A\mathrm{MTP}}\ (\|)$ respectively. Here, we see that $V_{A\mathrm{MTP}}\ (\perp)$ contains the thermoelectric response that is introduced due to the longitudinal thermal asymmetry that is induced by the presence of the notch. In the case of the Bloch domain wall, it is interesting to note that by inspection of Eq. 4 (main text) we can see that as there is no magnetisation present in the yz-plane the response is identical to the case of the saturated magnetic state of the microwire. Fig. S7 (a) ii, highlights that $V_{\mathrm{AMTP}}\ (\|)$ contains no thermoelectric response as we discuss in greater detail in the main text. Fig. S7 (b) i-iii, shows line profiles of the evolution of the two component responses as we start from a vertical offset of $600\ nm$ in b (i) and move progressively closer to the notch region in b (ii) at $50\ nm$ from the notch centre. Here, $V_{\mathrm{AMTP}}\ (\perp)$ increases in magnitude as we approach the notch region reaching a maximum in signal close to the notch centre. $V_{\mathrm{AMTP}}(\perp)$ remains approximately zero as no net thermoelectric response will arise for the Bloch domain wall.

Néel wall: Fig. S8 again shows the thermoelectric response for the separated AMTP Seebeck components, this time for the Néel domain wall. Fig. S8 (a) i-ii shows the perpendicular and parallel AMTP Seebeck voltage $V_{\mathrm{AMTP}}\ (\perp)$ and $V_{\mathrm{AMTP}}\ (\|)$ respectively. Again, we see that $V_{\mathrm{AMTP}}\ (\perp)$ contains the thermoelectric response that is introduced due to the longitudinal thermal asymmetry that is introduced into the notched region described in S2 of the SI. The response is approximately equivalent to that of the Bloch domain wall indicating that $V_{A\mathrm{MTP}}\ (\perp)$ predominantly arises due to the geometric asymmetry in the notch. $V_{A\mathrm{MTP}}\ (\|)$ now contains the response of the Néel domain wall as discussed in detail in the main text. The line profiles, Fig. S8 (b) i-iii, show very similar responses for $V_{\mathrm{AMTP}}\ (\perp)$ as those presented in Fig. S8 (b) i-iii, again highlighting the effect of the thermal asymmetry in the notched region. However, $V_{\mathrm{AMTP}}\ (\|)$ now shows the response from the Néel domain wall, which is opposite in sign to that introduced by $V_{\mathrm{AMTP}}\ (\perp)$ and reaches a maximum close to the notch centre.

Néel-Bloch wall comparison: In Figure S9 (a) (i-iii) we plot the calculated $V_{\mathrm{AMTP}}\ (\perp)$ for both wall types from Figure S8(a) to extract any subtle differences. Here, we show that starting from the line profiles closest to the notch, 50nm Fig. S9 (a) i, we observe that there is a marginal difference between the two data sets. This reduces with increasing distance from the notch, 300nm and 600nm (Fig. S9 (a) ii-iii, respectively). The subtracted difference between $V_{\mathrm{AMTP}}\ (\perp)$ for the two wall types are plotted in Fig. S9 (b) i-iii. Again, starting from the line profile closet to the notch, 50nm in Fig. S9 (b) (i), we observe the largest change of the order of $0.4\ \mu V$.

Inspection of Eq. 4, Table 1 in the main text, reveals that for perpendicular Seebeck component we should additionally see a response from magnetisation components in the yz-plane. For the Néel domain wall, this results in a small additional signal which superimposes with the thermal asymmetry response due to the notch. With increasing distance from the notch, the influence of both the thermal asymmetry and the Néel domain wall decays. $300\ nm$ from the notch the response is already reduced to $0.025\ \mu V$, Fig. S9 (b) ii, and at $600\ nm$ the response is further reduced to $0.002\ \mu V$, Fig. S9 (b) iii. These results

demonstrate that the main difference between the Néel and Bloch domain walls come from the AMTP, as indicated in Fig. S9.

5. Landau-Lifshitz-Bloch-based temperature-dependent micromagnetic model

This section gives more details of the micromagnetic model used in the calculations. As mentioned in the main article, the equation of motion describing the time-evolution of the magnetic moments is the Landau-Lifshitz-Bloch (LLB) equation Eq. 18 which is now well documented in the literature [33, 34, 53, 54]. The LLB equation is more suitable for temperature-dependent modelling since the traditional LLG equation conserves the magnetisation modulus, which is not applicable in our measurement system.

The LLB equation was originally derived by Garanin [53] within a mean-field approximation from the classical Fokker-Planck equation for atomistic spins interacting with a heat bath and can be written:

$$\dot{\boldsymbol{m}} = -\gamma \boldsymbol{m}_i \times \boldsymbol{H}_i^{eff} + \frac{\gamma\alpha_{\parallel}}{m^2}\left(\boldsymbol{m}\cdot\boldsymbol{H}_i^{eff}\right)\boldsymbol{m} - \frac{\gamma\alpha_{\perp}}{m^2}\left[\boldsymbol{m}\times\left(\boldsymbol{m}\times\boldsymbol{H}_i^{eff}\right)\right] \quad (18)$$

where **m** is a reduced magnetization $(M/M_s V)$, with $M_s$being the saturation magnetization at given temperature T and V is the volume of the micromagnetic cell. The first term in the expression is the usual adiabatic precession term, whilst the second and third terms are responsible for the relaxation of the modulus of the magnetization and transverse damping, respectively. The transverse and longitudinal relaxation parameters, $\alpha_{\perp}$ and $\alpha_{\parallel}$ respectively, determine the rate at which the motion of the magnetization is damped given by:

$$\alpha_{\perp} = \lambda\left(1 - \frac{T}{3T_c}\right) \quad (19)$$

$$\alpha_{\parallel} = \frac{2\lambda T}{3T_c} \quad (20)$$

The Curie temperature was $T_C = 580\ K$, and λ is a coupling parameter that describes the rate of transfer of energy out of the system.

The effective field in the LLB equation is defined as:

$$\boldsymbol{H}^{eff} = \boldsymbol{H}^{exc} + \boldsymbol{H}^{ani} + \boldsymbol{H}^{d} + \boldsymbol{H}^{ext} + \frac{1}{2\chi_{\parallel}}\left(1 - \frac{m^2}{m_e^2}\right)\boldsymbol{m} \quad (21)$$

where m_e is the equilibrium reduced magnetisation $\mathrm{m_e} = \frac{\mathrm{M_s(T)}}{\mathrm{M_s(0)}}$. The function $m_e(T)$ was fitted to experimental measurements using the following expression [55] up to $p = 9$:

$$m_e(T) = s_1\left[\sum_{p=1}^{9} a_p\left(\frac{T_c - T^p}{T}\right)\right] + c_1, T < T_c \quad (22)$$

$\widetilde{\chi_{||}}$ is the longitudinal susceptibility, described by:

$$\widetilde{\chi_{||}} = s_1\left[a_0\frac{T_c}{T_c - T} + \sum_{p=1}^{9} a_p(T_c - T)^p\right] + c_1, T < T_c \quad (23)$$

The first term in the field Eq. (19) corresponds to the exchange field:

$$\boldsymbol{H}_{exc} = \frac{2A(T)}{m_e^2 M_s(0)\Delta^2} \sum_{j \in nn} (\boldsymbol{m}_j - \boldsymbol{m}_i) \tag{24}$$

where A is the exchange stiffness constant, Δ is the contact area between the neighbouring discretization units (cubic cells). The micromagnetic parameters are temperature-dependent following the scaling with magnetisation relations. For the exchange parameter we assume that of cobalt [58]

$$A(T) = A(0) \times m^{1.76} \tag{25}$$

The anisotropy constant, K_1^u is assumed to be uniaxial and perpendicular to the plane. Its temperature dependence follows the Callen-Callen law [55]

$$K(T) = K(0) \times m^3 \tag{26}$$

The values of the micromagnetic parameters are given in Table S2.

Following the standard micromagnetic approach but with variable magnetisation length, the demagnetising field acting on the site *i* is calculated as:

$$\boldsymbol{H}_{d,i} = -M_s \sum_j \widehat{\boldsymbol{N}}(r_i - r_j) \cdot \boldsymbol{m}_j \tag{27}$$

where $\widehat{\boldsymbol{N}}$ is a 3x3 symmetric demagnetizing tensor. The sum runs over all cells at position $\boldsymbol{r}_{i,j}$. The demagnetising tensor is given by:

$$\widehat{\boldsymbol{N}}(\boldsymbol{r}_i - \boldsymbol{r}_j) = \frac{1}{4\pi} \oint \oint \frac{\mathrm{d}\boldsymbol{S}_i \mathrm{d}\boldsymbol{S}_j}{|\boldsymbol{r} - \boldsymbol{r}'|} \tag{28}$$

where $S_i(S_j)$ are the surface of the cell i (j) respectively, *r* and *r*' are the points on the respective surfaces. For efficient calculation of the long-range demagnetising field, we calculate the convolution in Fourier space (for details see Ref. [57]) The integrals in Eq. 28 are calculated analytically using the approach of Newell [58]. The numerical integration of the LLB equation is performed via use of the Heun scheme with a timestep of 5 fs.

The above constitutes a micromagnetic framework within the LLB approach. In our full modelling, we discretise a stripe of $1500 \times 500 \times 1\ nm,$ with a V-notch of dimensions $870\ nm$ high and approximately $75\ nm$ wide at the narrowest section, where the discretisation was $5 \times 5 \times 1\ nm$ voxels. For each tip position, the temperature map is taken from thermal modelling. In each discretisation site, micromagnetic parameters are taken as corresponding to a local temperature, and the set of coupled LLB equations is integrated as a function of time. We present examples of micromagnetic configuration at different time shots for several tip positions in Fig. S10.

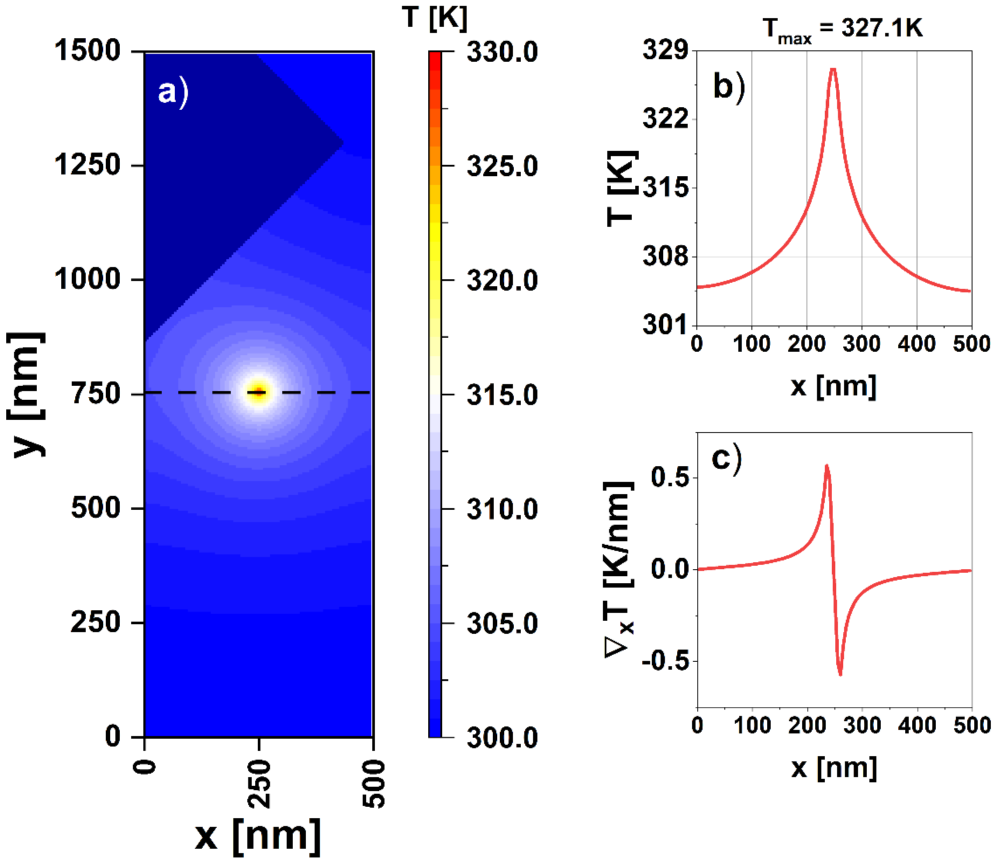

Fig. S1. Heatmap produced by the heat spot: (a) Thermal map when heat spot is far from the notch. **(b)** Profile line across the heat spot along x-axis depicted by the dashed line in (a). **(c)** Thermal gradient in the x-direction along the same line.

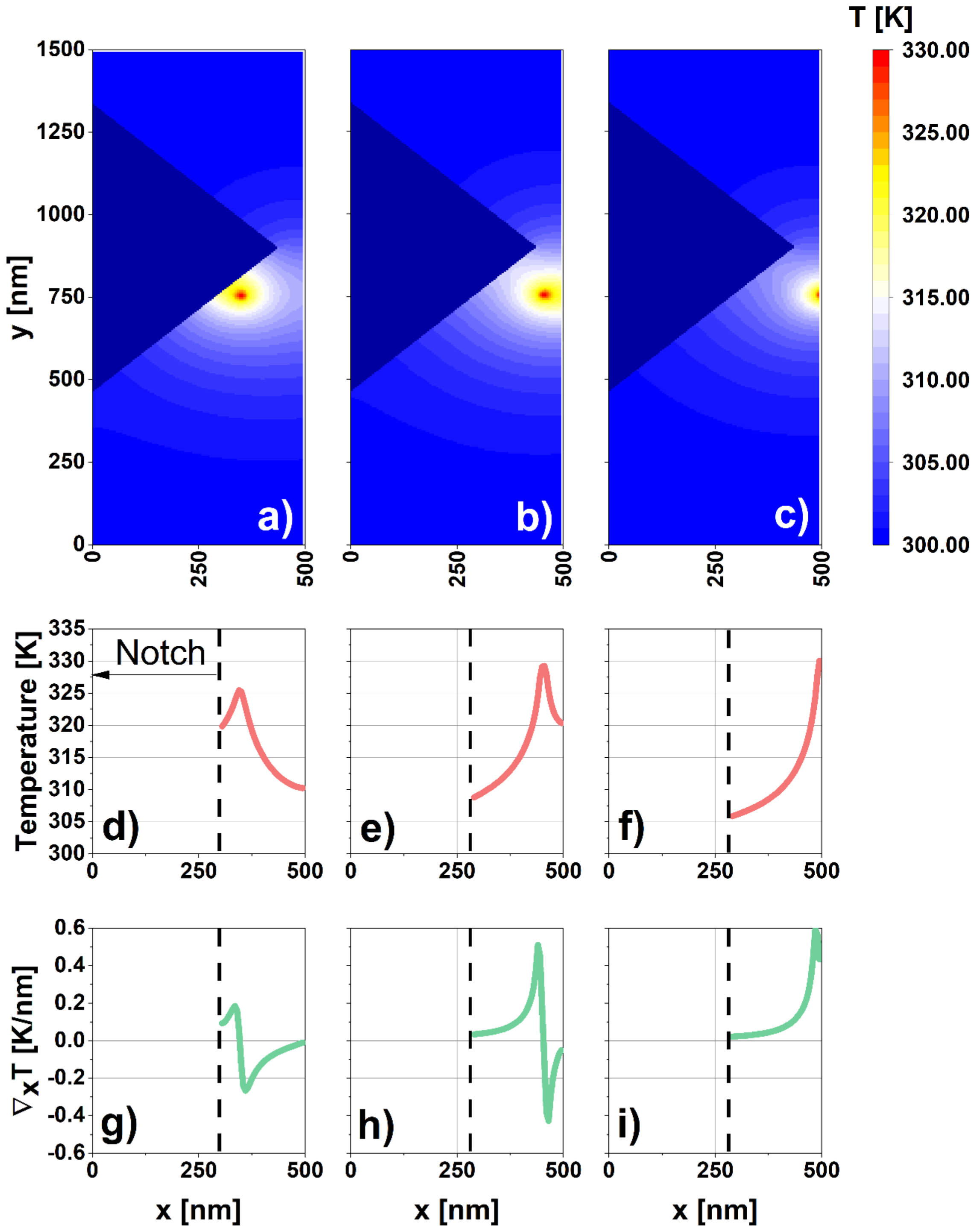

Fig. S2. (a-c) Heatmap for different heat spot positions across the wire. **(d-f)** Temperature profile for heatmaps (a-c) respectively, along the maximum of the heat spot in x-direction. **(g-i)** Thermal gradients corresponding to (d-f) in x-direction. The dashed line in (d-f) and (g-i) depicts the notch position.

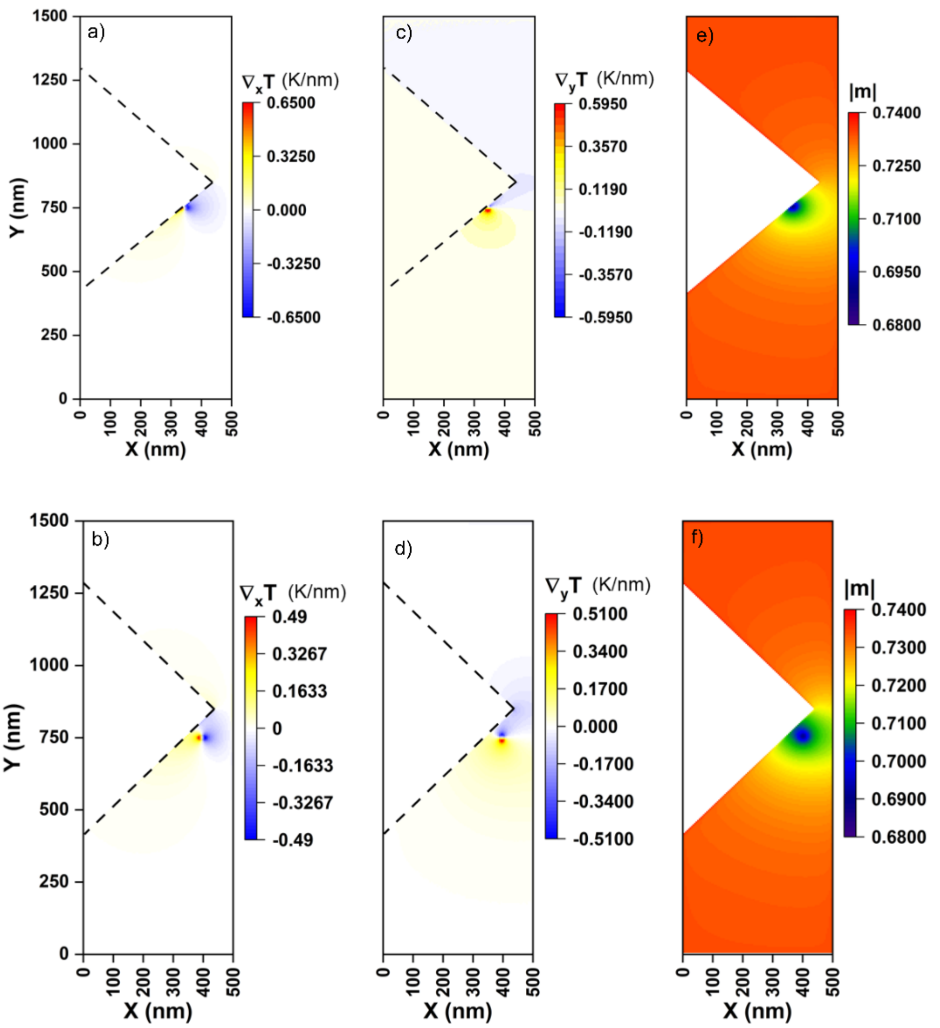

Fig. S3. Comparison of thermal gradient along x and y directions and local magnetisation in the vicinity of the probe: (a-d) Thermal gradients in x and y-direction when the heat spot is close to the notch edge (a-c) and at a location further from it (b-d). In these regions the thermal asymmetry appears and leads to non-zero thermoelectric contributions. **(e-f)** A reduction of magnetisation modulus is produced by the heat spot (calculations performed for $T_C = 580\ K$).

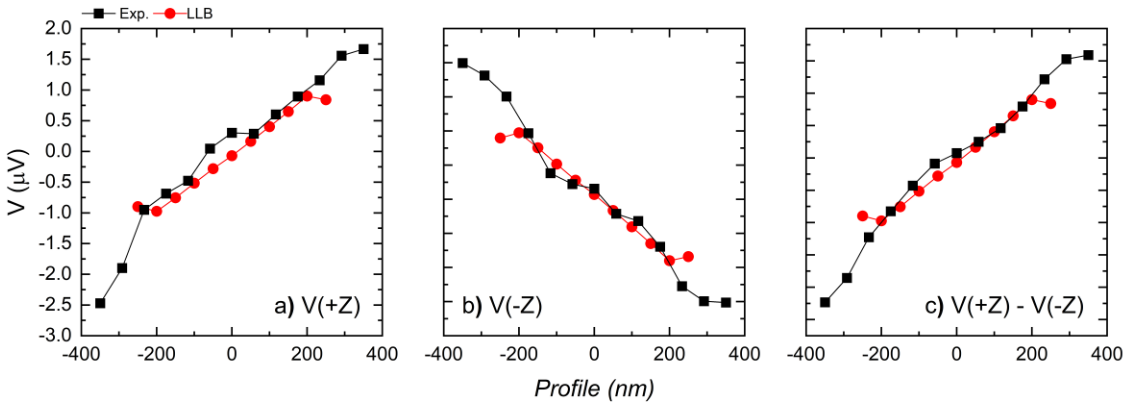

Fig. S4. Estimation of the product of $S_N \cdot T_{max}$ from experimental data far away from notch. (a) Sample saturated in +Z-direction. **(b)** Sample saturated in -Z-direction. **(c)** The sum of voltages V(+Z) - V(-Z) expression of voltage. Black squares correspond to experimental data while red circles correspond to the modelled data. (Profiles along x-direction.)

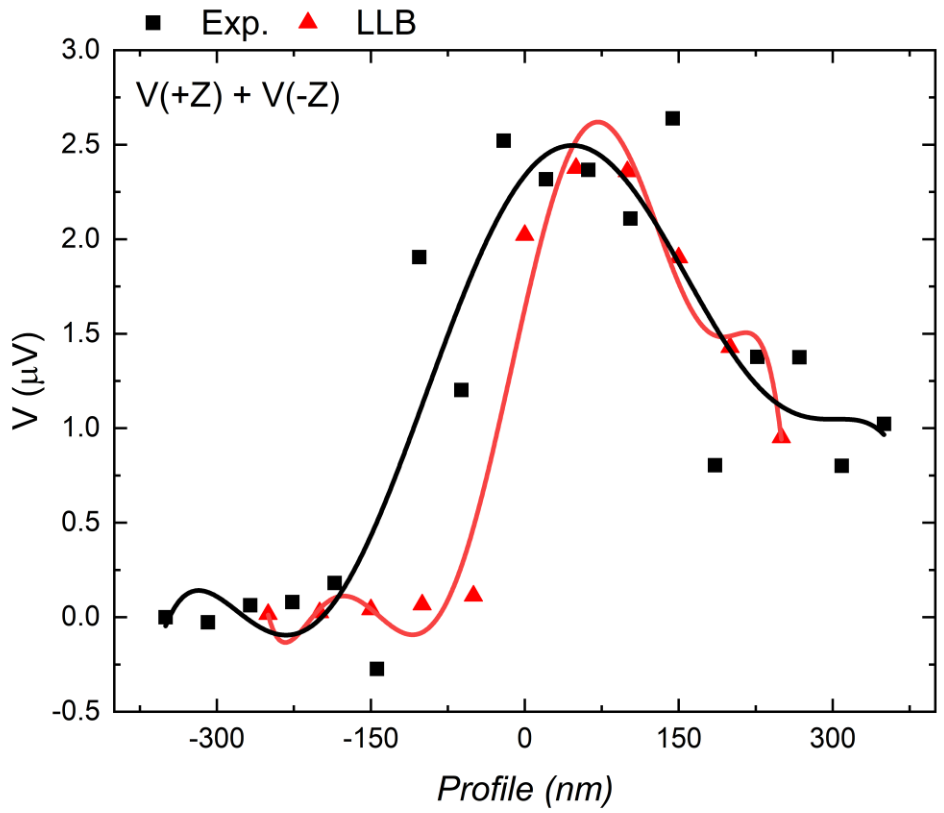

Fig. S5. The sum voltages for samples saturated in +Z and -Z directions at $200\ nm$ distance from the notch (used to fit the perpendicular Seebeck coefficients). Black squares correspond to experimental data while red triangles correspond to the modelled data. (Profiles along x-direction.)

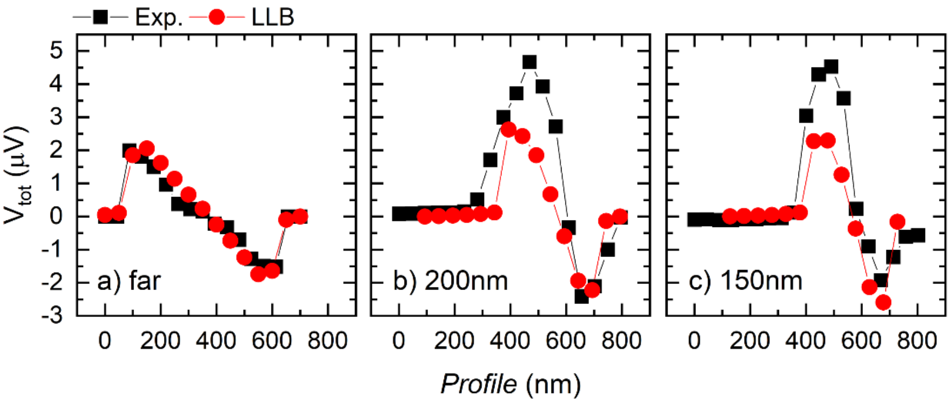

Fig. S6. Comparison between simulated and experimental voltage data: a) Far away from the notch **b)** 200nm below the notch. **c)** 150nm below the notch. The parameters corresponded to $\boldsymbol{T_{max} = 327\ K}$.

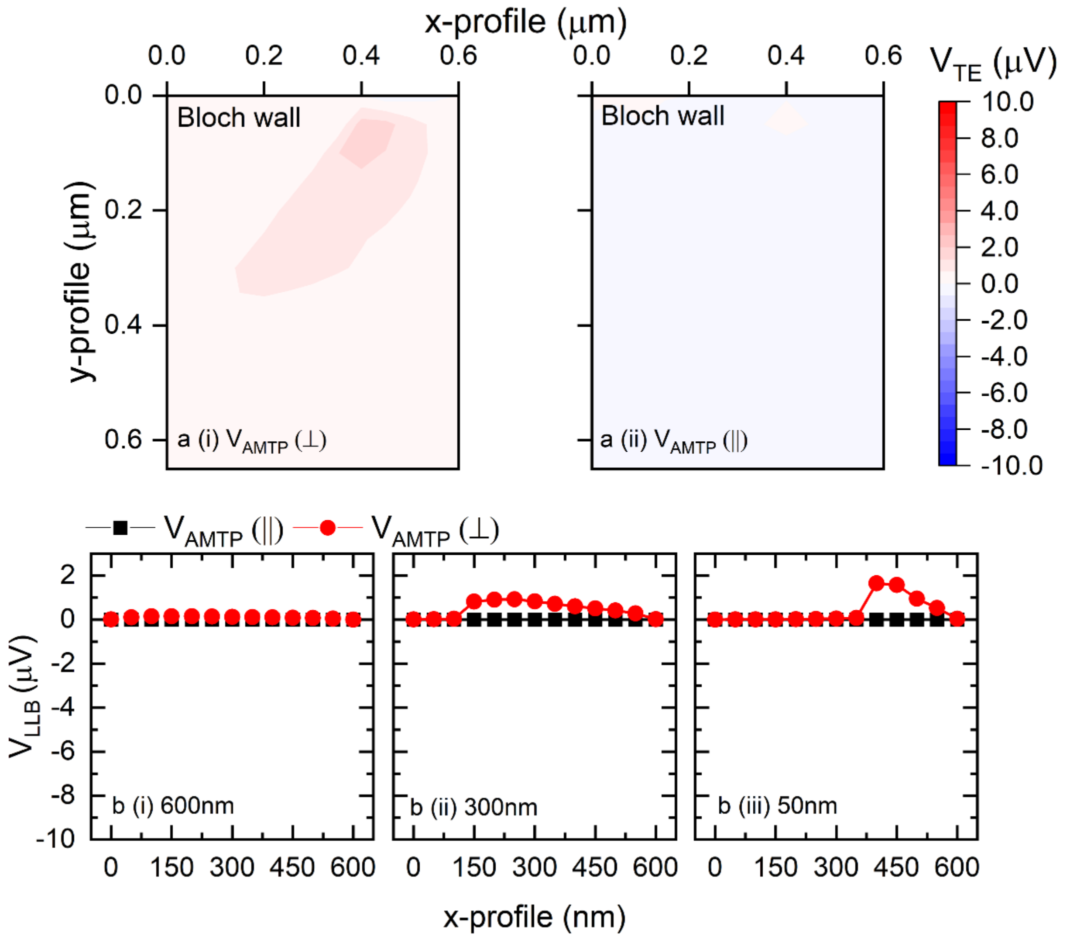

Fig. S7. Separated AMTP response evaluated from the analytical Bloch domain wall at the notch centre. a) (i) perpendicular AMTP Seebeck response highlighting the thermoelectric signal that arises from the broken thermal asymmetry in the vicinity of the notch regions; a) (ii) parallel AMTP Seebeck response showing no net signal due to the absence of the required plane of rotation for the spins in the domain wall. **b)** (i-iii) displays line profiles for the separated AMTP Seebeck components at various vertical offsets spanning 600 nm to 50 nm from the notch centre respectively.

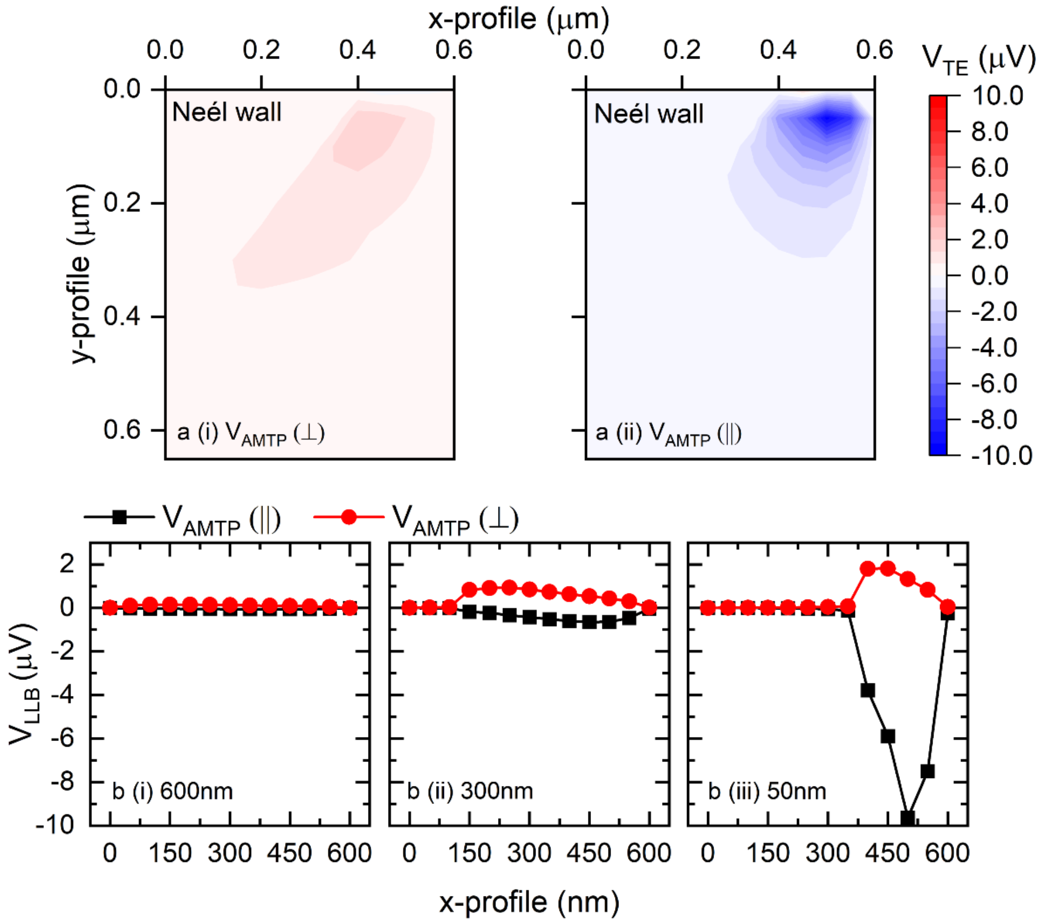

Fig. S8. Separated AMTP response evaluated from the analytical Néel domain wall at the notch centre. a) (i) perpendicular anisotropic magnetothermopower Seebeck response highlighting the thermoelectric signal that arises from the broken thermal asymmetry in the vicinity of the notch regions; a) (ii) parallel anisotropic magnetothermopower Seebeck response showing no net signal due to the absence of the required plane of rotation for the spins in the domain wall. **b)** (i-iii) displays line profiles for the separated anisotropic thermopower Seebeck components at vertical offsets 600 nm, 300 nm and 50 nm from the notch centre, respectively.

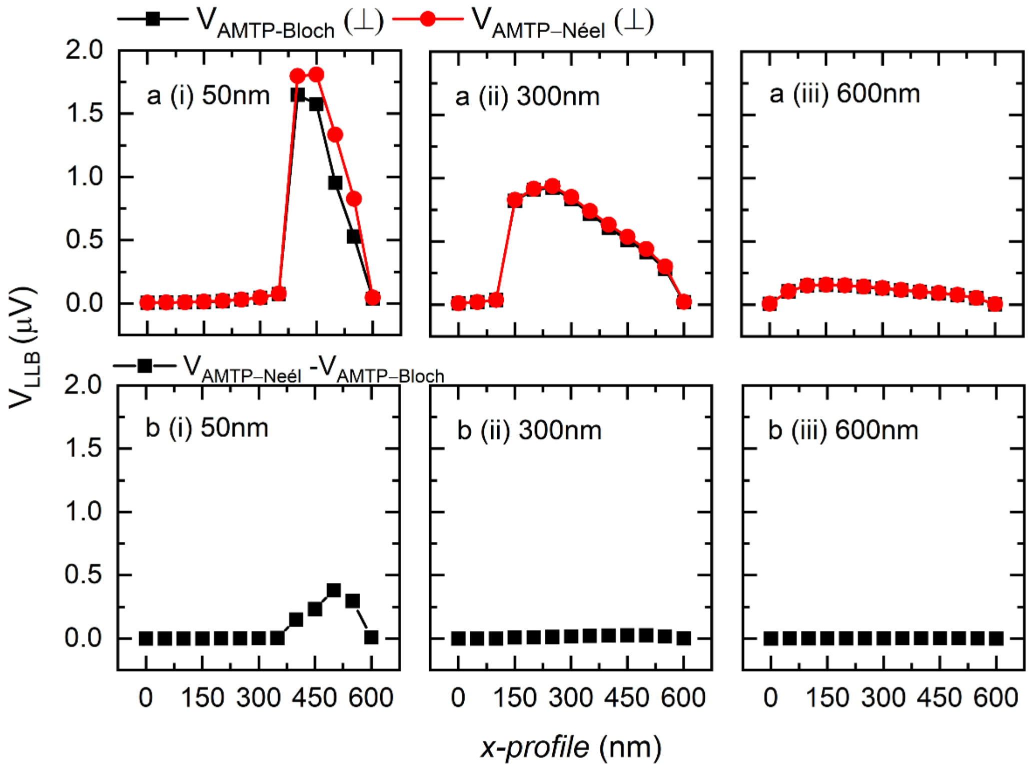

Fig. S9. Line profile comparison of V_{AMTP} (⊥) for the Bloch and Néel domain wall located at the notch centre. a) (i) line profile at 50nm from the notch centre demonstrating a small difference between the Bloch and Néel domain walls; a) (ii) the same at 300nm from the notch centre. Here the small differences are significantly reduced; and a) (iii) at 600nm the difference is almost zero. The difference, $\boldsymbol{V_{AMTP-N\acute{e}el}}$ (⊥) $-$ $\boldsymbol{V_{AMTP-Bloch}}$ (⊥), is plotted explicitly in **b)** (i-iii) where the maximum difference is approximately $\mathbf{0.4}$ μ$\boldsymbol{V}$, $\mathbf{0.025}$ μ$\boldsymbol{V}$ and $\mathbf{0.002}$ μ$\boldsymbol{V}$ and $\mathbf{50}\ \boldsymbol{nm}$, $\mathbf{300}\ \boldsymbol{nm}$, and $\mathbf{600}\ \boldsymbol{nm}$ from the notch centre. The differences shown here, highlight the influence of the magnetisation in the Néel domain wall which is higher closer to the notch centre.

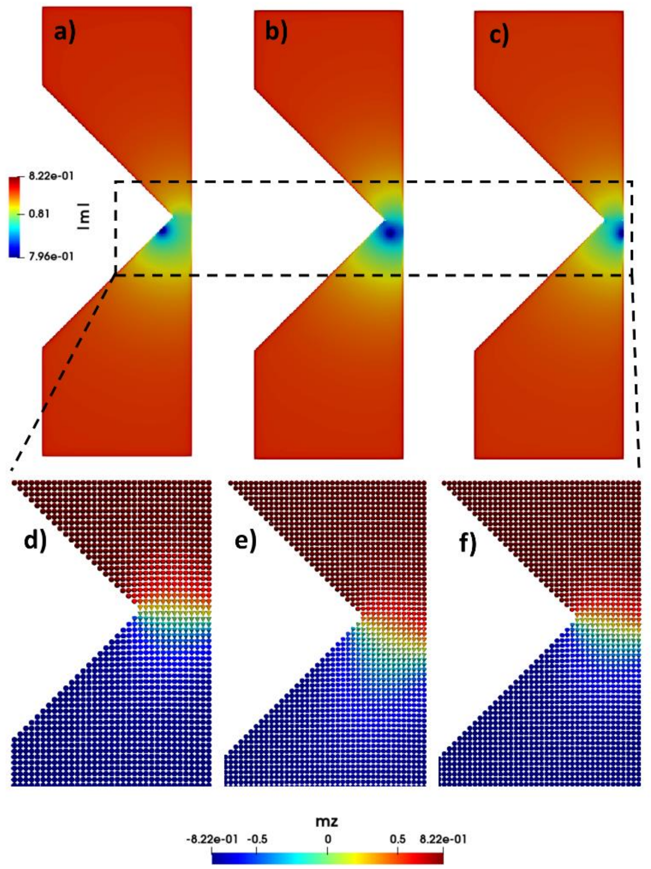

Fig. S10. Micromagnetic configuration at different time shots for several tip positions: (a-c) A reduction of magnetisation is produced by the heat spot. **(d-e)** Spin vector field for different heat spot positions. The heat spot produces a displacement of the domain wall towards hotter region. (Calculations performed at $T_C = 580\ K$)

Table S1. Maximum temperature, Nernst and Seebeck thermpower coefficients fitted from the experimental data.

T_{max} (K)	S_N (μV K^{-1})	$S_\perp$ (μV K^{-1})
333	-0.160	-0.063
327	-0.200	-0.079
320	-0.267	-0.105
316	-0.360	-0.140

Table S2. CoFeB material parameters used in the micromagnetic model based on the LLB equation.

Parameter	Value (units)
K_u	0.90e6 ($J\ m^{-3}$)
n	[001]
M_s	1.06e6 ($J\ T^{-1}\ m^{-3}$)
A	1.2e-11($J\ m^{-1}$)
T_C	580 (K)

REFERENCES AND NOTES

1. G. E. W. Bauer, E. Saitoh, B. J. van Wees, Spin caloritronics. *Nat. Mater.* **11**, 391–399 (2012).

2. B. Sothmann, R. Sanchez, A. N. Jordan, Thermoelectric energy harvesting with quantum dots. *Nanotechnology* **26**, 032001 (2015).

3. H. Yu, S. D. Brechet, J.-P. Ansermet, Spin caloritronics, origin and outlook. *Phys. Lett. A*, **381**, 825–837 (2017).

4. S. Bader, S. Parkin, Spintronics. *Annu. Rev. Condens. Matter Phys.* **1**, 71–88 (2010).

5. J. Wells, P. Krzysteczko, A. Caprile, B. Gribkov, H. W. Schumacher, J. H. Lee, R. Cowburn, O. Kazakova, Magnetic particle nanosensing by nucleation of domain walls in ultra-thin CoFeB/Pt devices. *IEEE Trans. Magn.* **52**, 1–5 (2016).

6. C. Vautrin, M. Vyazmensky, S. Engel, S. M. Murtry, M. Hehn, F. Montaigne, D. Lacour, R. S. Marks, Biochip based on arrays of switchable magnetic nano-traps. *Sens. Actuators B*, **251**, 699–705 (2017).

7. S. R. Boona, R. C. Myersbc, J. P. Heremans, Spin caloritronics. *Energ. Environ. Sci.* **7**, 885 (2014).

8. K.-I. Uchida, S. Takahashi, K. Harii, J. Ieda, W. Koshibae, K. Ando, S. Maekawa, E. Saitoh, Observation of the spin Seebeck effect. *Nature* **455**, 778–781 (2008).

9. H. Adachi, K.-I. Uchida, E. Saitoh, S. Maekawa, Theory of the spin Seebeck effect. *Rep. Prog. Phys.* **76**, 036501 (2013).

10. S. Daimon, R. Iguchi, T. Hioki, E. Saitoh, K. I. Uchida, Thermal imaging of spin Peltier effect. *Nat. Commun.* **7**, 13754 (2016).

11. Y. Ohnuma, M. Matsuo, S. Maekawa, Theory of the spin Peltier effect. *Phys. Rev. B Condens. Matter* **96**, 134412 (2017).

12. S. Meyer, Y. T. Chen, S. Wimmer, M. Althammer, T. Wimmer, R. Schlitz, S. Geprägs, H. Huebl, D. Ködderitzsch, H. Ebert, G. E. W. Bauer, R. Gross, S. T. B. Goennenwein, Observation of the spin Nernst effect, *Nat. Mater.* **16**, 977–981 (2017).

13. Y. Onose, T. Ideue, H. Katsura, Y. Shiomi, N. Nagaosa, Y. Tokura, Observation of the magnon Hall effect. *Science*, **329**, 297–299 (2010).

14. P. W. Bridgman, The connections between the four transverse galvanomagnetic and thermomagnetic phenomena. *Phys. Rev.* **24**, 644–651 (1924).

15. W. Nernst, Ueber die electromotorischen Kräfte, welche durch den Magnetismus in von einem Wärmestrome durchflossenen Metallplatten geweckt werden. *Ann. Phys. Chem.* **267**, 760–789 (1887).

16. D. Meier, D. Reinhardt, M. Schmid, C. H. Back, J.-M. Schmalhorst, T. Kuschel, G. Reiss, Influence of heat flow directions on Nernst effects in Py/Pt bilayers. *Phys. Rev. B Condens. Matter* **88**, 184425 (2013).

17. P. Krzysteczko, J. Wells, A. Fernandez-Scarioni, Z. Soban, T. Janda, X. Hu, V. Saidl, R. P. Campion, R. Mansell, J.-H. Lee, R. P. Cowburn, P. Nemec, O. Kazakova, J. Wunderlich, H. W. Schumacher, Nanoscale thermoelectrical detection of magnetic domain wall propagation. *Phys. Rev. B Condens. Matter* **95**, 220410 (2017).

18. T. Liang, J. Lin, Q. Gibson, T. Gao, M. Hirschberger, M. Liu, R. J. Cava, N. P. Ong, Anomalous Nernst effect in the dirac semimetal Cd_3As_2. *Phys. Rev. Lett.* **118**, 136601 (2017).

19. A. Sakai, Y. P. Mizuta, A. A. Nugroho, R. Sihombing, T. Koretsune, M. T. Suzuki, N. Takemori, R. Ishii, D. Nishio-Hamane, R. Arita, P. Goswami, S. Nakatsuji, Giant anomalous Nernst effect and quantum-critical scaling in a ferromagnetic semimetal. *Nat. Phys.* **14**, 1119–1124 (2018).

20. U. Martens, T. Huebner, H. Ulrichs, O. Reimer, T. Kuschel, R. R. Tamming, C.-L. Chang, R. I. Tobey, A. Thomas, M. Münzenberg, J. Walowski, Anomalous Nernst effect and three-dimensional temperature gradients in magnetic tunnel junctions. *Commun. Phys.* **1**, 65 (2018).

21. Z. Wang, M. Guo, H. A. Zhou, L. Zhao, T. Xu, R. Tomasello, H. Bai, Y. Dong, S.-G. Je, W. Chao, H.-S. Han, S. Lee, K.-S. Lee, Y. Yao, W. Han, C. Song, H. Wu, M. Carpentieri, G. Finocchio, M.-Y. Im, S.-Z. Lin, W. Jiang, Thermal generation, manipulation and thermoelectric detection of skyrmions. *Nat. Electron.* **3**, 672–679 (2020).

22. A. Fernández Scarioni, C. Barton, H. Corte-León, S. Sievers, X. Hu, F. Ajejas, W. Legrand, N. Reyren, V. Cros, O. Kazakova, H. W. Schumacher, Thermoelectric signature of individual skyrmions. *Phys. Rev. Lett.* **126**, 077202 (2021).

23. A. Sola, V. Basso, M. Kuepferling, C. Dubs, M. Pasquale, Experimental proof of the reciprocal relation between spin Peltier and spin Seebeck effects in a bulk YIG/Pt bilayer. *Sci. Rep.* **9**, 2047 (2019).

24. J. M. Bartell, C. L. Jermain, S. V. Aradhya, J. T. Brangham, F. Yang, D. C. Ralph, G. D. Fuchs, Imaging magnetization structure and dynamics in ultrathin $Y_3Fe_5O_{12}$/Pt bilayers with high sensitivity using the time-resolved longitudinal spin Seebeck effect. *Phys. Rev. Appl.* **7**, 044004 (2017).

25. I. Gray, G. M. Stiehl, J. T. Heron, A. B. Mei, D. G. Schlom, R. Ramesh, D. C. Ralph, G. D. Fuchs, Imaging uncompensated moments and exchange-biased emergent ferromagnetism in FeRh thin films. *Phys. Rev. Mater.* **3**, 124407 (2019).

26. A. B. Mei, I. Gray, Y. Tang, J. Schubert, D. Werder, J. Bartell, D. C. Ralph, G. D. Fuchs, D. G. Schlom, Local photothermal control of phase transitions for on-demand room-temperature rewritable magnetic patterning. *Adv. Mater.* **32**, 2001080 (2020).

27. J. M. Bartell, D. H. Ngai, Z. Leng, G. D. Fuchs, Towards a table-top microscope for nanoscale magnetic imaging using picosecond thermal gradients. *Nat. Commun.* **6**, 8460 (2015).

28. E. Pfitzner, X. Hu, H. W. Schumacher, A. Hoehl, D. Venkateshvaran , M. Cubukcu, J.-W. Liao, S. Auffret, J. Heberle, J. Wunderlich, B. Kästner, Near-field magneto-caloritronic nanoscopy on ferromagnetic nanostructures. *AIP Adv.* **8**, 125329 (2018).

29. T. Janda, J. Godinho, T. Ostatnicky, Pfitzner, G. Ulrich, A. Hoehl, S. Reimers, Z. Šobáň, T. Metzger, H. Reichlová, V. Novâk, R. P. Campion, J. Heberle, P. Wadley, K. W. Edmonds, O. J. Amin, J. S. Chauhan, S. S. Dhesi, F. Maccherozzi, R. M. Otxoa, P. E. Roy, K. OlejnÍk, P. Němec, T. Jungwirth, B.

Kaestner, J. Wunderlich, Magneto-Seebeck microscopy of domain switching in collinear antiferromagnet CuMnAs. *Phys. Rev. Mater.* **8**, 094413 (2020).

30. A. Sola, C. Barton, V. Basso, C. Dubs, M. Pasquale, O. Kazakova, Local spin Seebeck imaging with a scanning thermal probe. *Phys. Rev. Appl.* **14**, 034056 (2020).

31. A. Harzheim, C. Evangeli, O. Kolosov, P. Gehring, Local spin Seebeck imaging with a scanning thermal probe. *2D Mater.* **7**, 041004 (2020).

32. U. Atxitia, D. Hinzke, U. Nowak, Fundamentals and applications of the Landau–Lifshitz–Bloch equation. *J. Phys. D Appl. Phys.* **50**, 033003 (2017).

33. O. Chubykalo-Fesenko, U. Nowak, R. W. Chantrell, D. Garanin, Dynamic approach for micromagnetics close to the Curie temperature. *Phys. Rev. B* **74**, 094436 (2006).

34. N. Kazantseva, D. Hinzke, U. Nowak, R. W. Chantrell, U. Atxitia, O. Chubykalo-Fesenko, Towards multiscale modeling of magnetic materials: Simulations of FePt. *Phys. Rev. B* **77**, 184428 (2008).

35. Y. Dovzhenko, F. Casola, S. Schlotter, T. X. Zhou, F. Büttner, R. L. Walsworth, G. S. D. Beach, A. Yacoby, Magnetostatic twists in room-temperature skyrmions explored by nitrogen-vacancy center spin texture reconstruction. *Nat. Commun.* **9**, 2712 (2018).

36. O. Reimer, D. Meier, M. Bovender, L. Helmich, J.-O. Dreessen, J. Krieft, A. S. Shestakov, C. H. Back, J.-M. Schmalhorst, A. Hütten, G. Reiss, T. Kuschel, Quantitative separation of the anisotropic magnetothermopower and planar Nernst effect by the rotation of an in-plane thermal gradient. *Sci. Rep.*, **7**, 40586 (2017).

37. N. Nagaosa, J. Sinova, S. Onoda, A. H. MacDonald, N. P. Ong, Anomalous Hall effect. *Rev. Mod. Phys.* **82**, 1539–1592 (2010).

38. M. V. Berry, Quantal phase factors accompanying adiabatic changes. *Proc. R. Soc. Lond.* **392**, 45–57 (1984).

39. L. Berger, Side-jump mechanism for the Hall effect of ferromagnets. *Phys. Rev. B* **2**, 4559–4566 (1970).

40. J. Smit, The spontaneous Hall effect in ferromagnetics I. *Physica* **21**, 877–887 (1955).

41. A. Campbell, A. Fert, O. Jaoul, The spontaneous resistivity anisotropy in Ni-based alloys. *J. Phys. C Solid State Phys.* **3,** S95–S101 (1970).

42. J. Bodzenta, A. Kaźmierczak-Bałata, K. Harris, Quantitative thermal measurement by the use of scanning thermal microscope and resistive thermal probes. *J. Appl. Phys.* **127**, 031103 (2020).

43. J. Duvigneau, H. Schönherr, G. J. Vancso, Nanoscale thermal AFM of polymers: Transient heat flow effects. *ACS Nano* **4**, 6932–6940 (2010).

44. X. S. Wang, X. R. Wang, Domain wall motion by localized temperature gradients. *Phys. Rev. B Condens. Matter*, **95**, 064419 (2017).

45. S. Moretti, V. Raposo, E. Martinez, L. Lopez-Diaz, Thermodynamic theory for thermal-gradient-driven domain-wall motion. *Phys. Rev. B Condens. Matter* **90**, 014414 (2014).

46. W. Wang, M. Albert, M. Beg, M.-A. Bisotti, D. Chernyshenko, D. Cortés-Ortuño, I. Hawke, H. Fangohr, Magnon-driven domain-wall motion with the Dzyaloshinskii-Moriya interaction. *Phys. Rev. B Condens. Matter* **114**, 087203 (2015).

47. M. T. Islam, X. S. Wang, X. R. Wang, Thermal gradient driven domain wall dynamics. *J. Phys. Condens. Matter*, **31**, 455701 (2019).

48. E. J. Torok, A. L. Olson, H. N. Oredson, Transition between Bloch and Néel Walls. *J. Appl. Phys.*, **36**, 1394–1399 (1965).

49. M. D. DeJong, K. L. Livesey, Analytic theory for the switch from Bloch to Néel domain wall in nanowires with perpendicular anisotropy. *Phys. Rev. B Condens. Matter*, **92**, 214420 (2015).

50. P. Asselin, R. F. L. Evans, J. Barker, R. W. Chantrell, R. Yanes, O. Chubykalo-Fesenko, D. Hinzke, U. Nowak, Constrained Monte Carlo method and calculation of the temperature dependence of magnetic anisotropy. *Phys. Rev. B Condens. Matter* **82**, 054415 (2010).

51. R. Moreno, R. F. L. Evans, S. Khmelevski, M. C. Munoz, R. W. Chantrell, O. Chubykalo-Fesenko, Temperature-dependent exchange stiffness and domain wall width in Co. *Phys. Rev. B Condens. Matter*, **94**, 104433 (2016).

52. P. Klapetek, J. Martinek, P. Grolich, M. Valtr, N. J. Kaur, Graphics cards based topography artefacts simulations in scanning thermal microscopy. *Int. J. Heat Mass Transf.* **108,** 841–850 (2017).

53. D. Garanin, Fokker-Planck and Landau-Lifshitz-Bloch equations for classical ferromagnets. *Phys. Rev. B* **55**, 3050–3057 (1997).

54. P. Nieves, O. Chubykalo-Fesenko, Modeling of ultrafast heat- and field-assisted magnetization dynamics in FePt. *Phys. Rev. Appl.* **5**, 014006 (2016).

55. H. Callen, E. Callen, The present status of the temperature dependence of magnetocrystalline anisotropy, and the power law. *J. Phys. Chem. Solid* **27**, 1271–1285 (1966).

56. R. Moreno, R. F. L. Evans, S. Khmelevskyi, M. C. Muñoz, R. W. Chantrell, O. Chubykalo-Fesenko, Temperature-dependent exchange stiffness and domain wall width in Co, *Phys. Rev. B* **94**, 104433 (2016).

57. T. A. Ostler, M. O. A. Ellis, D. Hinzke, U. Nowak, Temperature dependent ferromagnetic resonance via the Landau-Lifshitz-Bloch equation: Application to FePt. *Phys. Rev. B* **90**, 094402 (2014).

58. A. J. Newell, W. Williams, D. J. Dunlop, A generalization of the demagnetizing tensor for nonuniform magnetization. *J. Geophys. Res.* **98**, 9551–9555 (1993).
